# Pharyngeal carriage of *Neisseria* species in the African meningitis belt

**DOI:** 10.1016/j.jinf.2016.03.010

**Published:** 2016-06

**Authors:** Kanny Diallo, Caroline Trotter, Youssouf Timbine, Boubou Tamboura, Samba O. Sow, Bassira Issaka, Ibrahim D. Dano, Jean-Marc Collard, Marietou Dieng, Aldiouma Diallo, Adane Mihret, Oumer A. Ali, Abraham Aseffa, Stephen L. Quaye, Akalifa Bugri, Isaac Osei, Kadidja Gamougam, Lodoum Mbainadji, Doumagoum M. Daugla, Galadima Gadzama, Zailani B. Sambo, Babatunji A. Omotara, Julia S. Bennett, Lisa S. Rebbetts, Eleanor R. Watkins, Maria Nascimento, Arouna Woukeu, Olivier Manigart, Ray Borrow, James M. Stuart, Brian M. Greenwood, Martin C.J. Maiden

**Affiliations:** a Centre pour les Vaccins en Développement, Bamako, Mali; b Department of Veterinary Medicine, University of Cambridge, Cambridge, UK; c Centre de Recherche Médicale et Sanitaire, Niamey, Niger; d Institut de Recherche pour le Développement, Dakar, Senegal; e Armauer Hansen Research Institute, Addis Ababa, Ethiopia; f Navrongo Health Research Centre, Navrongo, Ghana; g Centre de Support en Santé International, N'Djamena, Chad; h University of Maiduguri, Nigeria; i Department of Zoology, University of Oxford, Oxford, UK; j London School of Hygiene & Tropical Medicine, London, UK; k Vaccine Evaluation Unit, Public Health England, Manchester, UK

**Keywords:** Non-meningococcal *Neisseria*, Pharyngeal carriage, African meningitis belt

## Abstract

**Objectives:**

*Neisseria meningitidis,* together with the non-pathogenic *Neisseria* species (NPNs), are members of the complex microbiota of the human pharynx. This paper investigates the influence of NPNs on the epidemiology of meningococcal infection.

**Methods:**

*Neisseria* isolates were collected during 18 surveys conducted in six countries in the African meningitis belt between 2010 and 2012 and characterized at the *rplF* locus to determine species and at the variable region of the *fetA* antigen gene. Prevalence and risk factors for carriage were analyzed.

**Results:**

A total of 4694 isolates of *Neisseria* were obtained from 46,034 pharyngeal swabs, a carriage prevalence of 10.2% (95% CI, 9.8–10.5). Five *Neisseria* species were identified, the most prevalent NPN being *Neisseria lactamica.* Six hundred and thirty-six combinations of *rplF*/*fetA_VR* alleles were identified, each defined as a *Neisseria* strain type. There was an inverse relationship between carriage of *N. meningitidis* and of NPNs by age group, gender and season, whereas carriage of both *N. meningitidis* and NPNs was negatively associated with a recent history of meningococcal vaccination.

**Conclusion:**

Variations in the prevalence of NPNs by time, place and genetic type may contribute to the particular epidemiology of meningococcal disease in the African meningitis belt.

## Highlights

• A prevalence of 10.2% of *Neisseria* infection was observed during the study.
• Five *Neisseria* species were identified in nasopharyngeal samples.
• High level of genetic diversity was observed in carried isolates.
• Inverse relationship between carriage of *Neisseria meningitidis* and non-pathogenic *Neisseria.*

## Introduction

The human pharynx hosts a complex microbiota, including bacteria belonging to the genus *Neisseria*. Most members of this genus are non-pathogenic commensals (non-pathogenic *Neisseria*, NPNs), which very rarely cause invasive disease, but *Neisseria meningitidis* (*Nm*), the meningococcus, is an exception.^1^ Despite advances in vaccine development, invasive meningococcal disease remains a public health challenge globally and especially in the African meningitis belt, where very large epidemics continue to occur,^2^, ^3^ despite the recent widespread deployment of a serogroup A meningococcal conjugate vaccine (PsA-TT, MenAfriVac^®^).^4^, ^5^, ^6^, ^7^, ^8^

Most pharyngeal carriage studies in the African meningitis belt have focused on the meningococcus,^9^, ^10^, ^11^, ^12^ with little attention paid to other *Neisseria* species apart from *Neisseria lactamica* (*Nl*). In northern Nigeria, pharyngeal carriage of *Nl* was common, especially in young children^13^ and genetic exchange among *Nm*, *Nl* and unspecified *Neisseria* species has been demonstrated in The Gambia.^14^ Molecular epidemiological studies of *Nm* and *Nl* conducted in Burkina Faso from 2009 to 2012 reported a high overall prevalence of *Nl* carriage (18.2%), a higher prevalence of *Nl* in males than in females, except in those aged 18–29 years, no change between dry and rainy season and no significant changes following vaccination with PsA-TT.^10^, ^15^, ^16^, ^17^ It has long been considered likely that carriage of NPNs influences host susceptibility to infection and invasion by *Nm,* a view supported by the fact that healthy subjects inoculated with *Nl* exhibit some protection against colonization with *Nm*.^18^ Until recently, *Nl* was considered to be the NPN genetically most similar to *Nm*. However, recent whole genome sequence (WGS) studies have shown that *Neisseria polysaccharea* (*Np*) and *Neisseria bergeri* (*Nb*) are more closely related to *Nm* than *Nl*.^19^ Consequently, these bacteria may also influence the epidemiology of *Nm* colonization and invasion. For this reason we have studied the prevalence of carriage with various *Neisseria* species in six countries of the African meningitis belt and investigated risk factors for their carriage.

## Materials and methods

The methods employed in the MenAfriCar surveys, during which the isolates described in this paper were collected, have been described in detail previously^11^ and are summarized briefly here. Ethical approval for the surveys was obtained from the ethics committee of the London School of Hygiene & Tropical Medicine and from ethical committees in each partner country. The study was registered with ClinicalTrials.gov (NCT01119482).

### Carriage surveys

**Figure 1:**
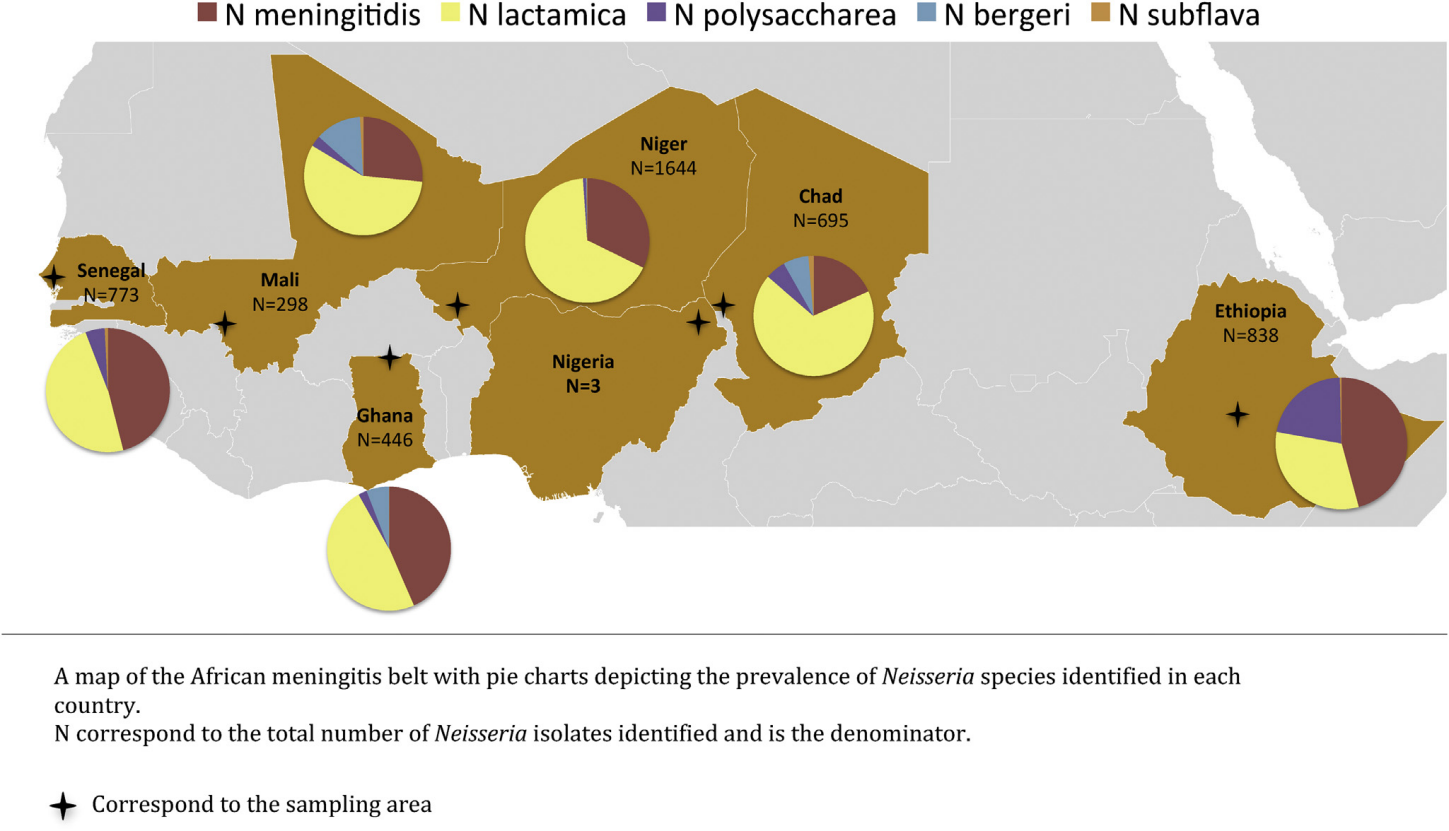
Frequency distribution of *Neisseria* species in each country of the study.

Bacteria were isolated during 18 cross-sectional surveys conducted in Chad, Ethiopia, Ghana, Mali, Niger and Senegal during 2010–2012 (Fig. 1). Pre-vaccination surveys in Mali, Niger and Chad had a target of 5000 participants and post-vaccination surveys a target of 2000 participants in Mali and Niger and 6000 in Chad. The remaining surveys aimed to recruit 2000 participants. A representative sample of households was obtained either from an updated demographic surveillance system (DSS) or from a census conducted for the study. Within selected households, individuals in four different age groups (0–4 years, 5–14 years, 15–29 years and 30 years or more) were chosen randomly until the required sample size was reached, with a maximum of 5 individuals recruited per household. Once written consent had been obtained, standardized household and individual questionnaires inquiring about risk factors for meningococcal infection were administered. Pharyngeal swabs were obtained using a standardized technique that involved swabbing both the posterior-pharyngeal wall and the tonsils.^20^

### Bacteriology

NPNs were isolated using the same conventional microbiology techniques that were employed for the detection of *Nm* described previously.^11^ Briefly, pharyngeal swabs were plated onto modified Thayer Martin agar plates and incubated 24–48 h at 37 °C in 5% CO_2;_ oxidase and gram stain testing identified oxidase positive, Gram-negative diplococci. Further biochemical tests (ortho-nitrophenyl-β-galactoside, ץ-glutamyl transpeptidase and tributyrin) were used in each site to differentiate between putative *Nl* and *Nm* isolates and members of the genus *Moraxella*.

### Molecular methods

A boiled cells suspension of each oxidase positive, Gram negative diplococci (OPGND) isolate was sent to the Department of Zoology at the University of Oxford for molecular typing, where Sanger sequencing was used to characterize gene targets as described in the Supplement. Sequences were assembled using SeqSphere (http://www.ridom.de/seqsphere/) and imported into the isolate's record previously created in a BIGSdb database.^21^

### *Neisseria* speciation

Amplification and sequencing of a 413 bp fragment of the *rplF* gene was used to differentiate among *Neisseria* species as described previously.^22^ A phylogeny based on the *f_rplF* alleles from Bennett et al. (2014) and the unique alleles found in this study was reconstructed using the Neighbor-Joining algorithm^23^ and the Kimura 2-parameter substitution model^24^ in mega version 6.0.^25^ For isolates that did not yield results for the *rplF* assay, sequencing of the *rrna* gene, encoding 16S rRNA,^26^ was used to confirm the presence or absence of a bacterium and to determine its genus. Only *rplF* confirmed NPNs were included in this study with the exception of four *Nm* speciated on the basis of the 16S rRNA and the *porA* sequences. New alleles were investigated by a BLAST of obtained sequences against the 16S rRNA sequence data of the EzTaxon server (http://www.ezbiocloud.net/eztaxon;^27^).

### Genetic diversity

Sequencing of the variable region of the *fetA* gene (*feta_VR*) was used to assess the genetic diversity of the *Neisseria* identified as described in the Supplementary methods.^28^ The sequences were assembled as for the other targets using SeqSphere (http://www.ridom.de/seqsphere/). The trace files of each new allele were manually curated in mega version 6.0,^25^ the quality of the sequences were assessed and the modified bases checked in both the forward and reverse trace file. The corresponding protein sequences were also aligned and compared with known sequences stored in the *Neisseria* sequence definition database of pubMLST^21^ before being entered into the allele database and the appropriate isolate record.

For isolates for which *fetA_VR* could not be amplified, an assay identifying the absence of the *fetA* gene (*fetA* null, *fnl*) was employed, using primers placed on genes on each side of the *fetA* gene: *thdF* and *fetB* as described in the Supplementary methods.^29^

### Statistical methods

Analyzes were performed using Stata v12.0 (StataCorp, Texas). Survey design and potential household clustering were taken into account using the survey commands in Stata. Carriage prevalence of each of the different *Neisseria* species, together with 95% confidence intervals, was calculated for each country and each survey. Risk factors for carriage of *Nm* and NPNs were assessed simultaneously using multinomial logistic regression. Each risk factor was considered in turn using univariable, multinomial logistic regression. A multivariable model was then constructed including country, age group and sex *a priori* and any variable with a p-value <0.1 in the univariable analyses; only the variables with a p value < 0.05 in the multivariable analysis were kept in the final model. As a final check, dropped variables were re-entered into the model one at a time and the p-values re-examined; the variable was retained as significant if the p value was <0.05.

## Results

### Prevalence of carriage with *Neisseria* species

A total of 4694 of the 46034 pharyngeal swabs collected yielded a *Neisseria* species, giving a carriage prevalence of 10.2% [95% CI, 9.8–10.5%]: 696 *Neisseria* were identified out of the 946 OPGND samples received from Chad; 838 out of the 994 received from Ethiopia; 446 out of the 544 received from Ghana; 298 out of the 504 from Mali; 1644 out of 2321 from Niger and 773 out of the 971 received from Senegal. The most frequently isolated species was *Nl* with a 5.6% point prevalence [95% CI, 5.3–5.8%], followed by *Nm* at 3.6% [95% CI, 3.4–3.8%]), *Np* at 0.6% [95% CI, 0.5–0.7%], *Nb* at 0.2% [95% CI, 0.2–0.3%] and *Neisseria subflava* (*Ns*) at 0.05% [95% CI, 0.03–0.1%] (Supplementary Table 1). Twenty isolates from Chad were identified as belonging to the *Neisseria* genus but did not cluster with any known species on the Neighbor-Joining Tree (NJT; data not shown); they were designated *Neisseria* sp. and will be investigated further by whole genome sequencing methods.

### Factors influencing the prevalence of carried *Neisseria* species

Country, season, age, gender, and a history of recent vaccination against *Nm* were associated with carriage of NPNs. Additionally, area of residence, household crowding and kitchen location were significant risk factors for carriage of *Nm* (Table 1).

*Country:* The prevalence of carriage of *Neisseria* species overall varied significantly among countries with Niger having the highest point prevalence (19.9% [95% CI, 18.9–20.9]), followed by Senegal (17.5% [95% CI, 16.1–18.8]), Ethiopia (13.8% [95% CI, 12.8–14.8]), Ghana (8.5%[95% CI, 7.6–9.4]), Chad (5.3%[95% CI, 4.9–5.7]) and Mali (3.4% [95% CI, 2.9–3.8]) (Supplementary Table 1). The distribution of the different species of *Neisseria* by country is shown in Fig. 1. The prevalence of *Nm* carriage varied significantly among countries, being highest in Senegal (8.0% [95% CI, 7.1–9.0]) and lowest in Mali (0.9% [95% CI, 0.7–1.1]). There were also major differences in the prevalence of carriage of NPNs by country and some NPNs were not identified in some countries, for example no *Nb* were isolated in Ethiopia and Senegal and no *Ns* in Ghana. Ethiopia was the only country where the prevalence of *Nm* carriage was higher than that of *Nl.*

*Year and season:* The prevalence of carriage of *Neisseria* species overall varied little over the three years of the study: 10.0% [95% CI, 9.4–10.5%] in the first survey, 11.1% [95% CI, 10.6–11.7%] in the second survey and 9.4% [95% CI, 8.9–9.9%] in the third survey (Table 2). The prevalence of carriage with both *Nm* and NPNs was also similar over time, with *Nl* being the most carried species, regardless of survey or season. There was, however, variation in the prevalence of carriage of NPNs over time at the country level: for example, there was an increase in the prevalence of *Nl* between surveys 1 and 2 in both Chad (from 1.1% to 5.9%) and Ghana (from 0.8% to 6.3%) and an increase in *Nm* prevalence between survey 2 and 3 in Senegal (from 3.1% to 19.8%^4^) (Supplementary Fig. 1). The relative risk of carriage of NPNs was significantly lower during the dry compared to the rainy season with an adjusted Relative Risk Ratio (aRRR) of 0.78, [95% CI, 0.70–0.86], whereas the opposite was true for *Nm* with an aRRR of 1.53, [95% CI, 1.35–1.74] in the dry season.

**Figure 2:**
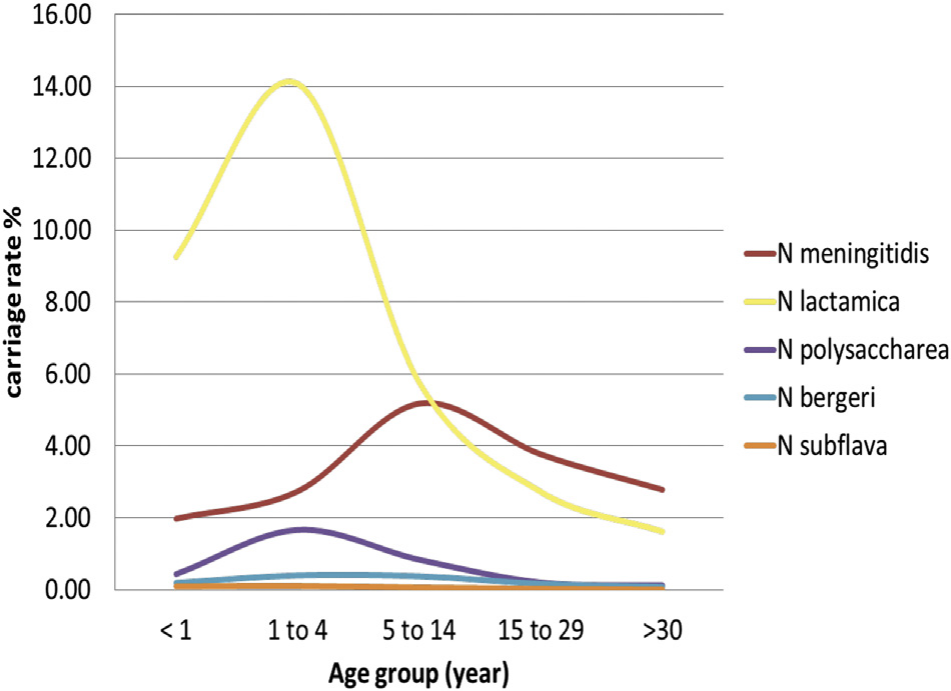
Prevalence of *Neisseria* species carried by age group.

*Age and gender*: The relative risk of carrying NPNs overall was higher in children aged less than 15 years compared to the young adult age group (age 15–29 years) with an aRRR of 3.11 [95% CI, 2.60–3.73] for those <1 year, 5.90 [95% CI, 5.23–6.65] for the 1–4 year olds and 2.59 (95% CI, 2.29–2.94) for the 5–14 year olds. The oldest age group >30 years had a lower aRRR of 0.51 (95% CI, 0.43–0.61). The prevalence of carried *Nl* was highest in the 1–4 year old age group, reaching a peak of 14.1% [95% CI, 13.3–14.8%] in contrast to carriage of *Nm,* which reached a peak of 5.2% [95% CI, 4.8–5.6%] in the 5–14 year age group. Similarly to *Nl, Np* carriage also reached a peak prevalence of 1.7% [95% CI, 1.4–1.9%] in the 1–4 year old age group (Fig. 2). Carriage prevalence of *Nb* and *Ns* was too low to identify an overall trend in age group distribution. Males had a lower risk of carrying NPNs than females (aRRR of 0.87 [95% CI, 0.80–0.94]) but a higher risk of carrying *Nm* (aRRR of 1.34 [95% CI, 1.21–1.48].

*Vaccination history*: A history of vaccination within the past 12 months with any meningococcal vaccine was associated with a decrease in *Nm* carriage, as reported previously,^8^ but additionally with a decreased risk of carrying NPNs (aRRR of 0.82 [95% CI, 0.73–0.92]. In the three countries where MenAfriVac^®^ was introduced during the course of the study (Chad, Mali, and Niger) a significant decrease in the prevalence of carriage of *Nl* from 6.4% to 4.9% was observed. An overall decrease in carriage of *Nm* was also observed in the three countries but this reduction was not consistent in all countries with Mali experiencing an increase from 0.6% to 1.2% (Supplementary Fig. 2).

*Other risk factors:* Area of residence (rural vs urban), crowding, cooking with cow dung or straw, kitchen location, and attendance at social gatherings in the past week all had a significant impact on the odds of carrying NPNs in the univariable regression model, but their effect was not significant in the multivariable model. Some of these risk factors were retained in the final model as, although they had no effect on NPN carriage, they were significant for *Nm* carriage.

### Genetic diversity of identified *Neisseria* species

Forty-two different alleles were identified for the *rplf* fragment (*f_rplf*). *Nl* was the most diverse species with 17 *f_rplf* alleles, the most frequent of which was *f_rplf 6* (1453 isolates, 56.1%). Eleven alleles were found for *Nm* the most frequent being *f_rplf 2* (749 isolates, 44.6%) and *f_rplf 1* (707 isolates, 42.1%); four *f_rplf* alleles were identified in *Np,* the most frequent of which was *f_rplf 9* (207 isolates, 71.4%). Four alleles were also found among the *Nb* alleles with *f_rplf 62* (57) and f_rplf 69 (46) representing 91.2% of these isolates. Finally, seven alleles were observed for *Ns*, with predominance of *f_rplf 43* (15 isolates, 62.5%) (Supplementary Table 2). A Neighbor Joining tree was used to represent the phylogeny of the *rplF* fragment present in the *Neisseria* isolates of this study in relation to the original isolates used to create the *f_rplF* assay^22^ (Supplementary Fig. 3).

**Figure 3:**
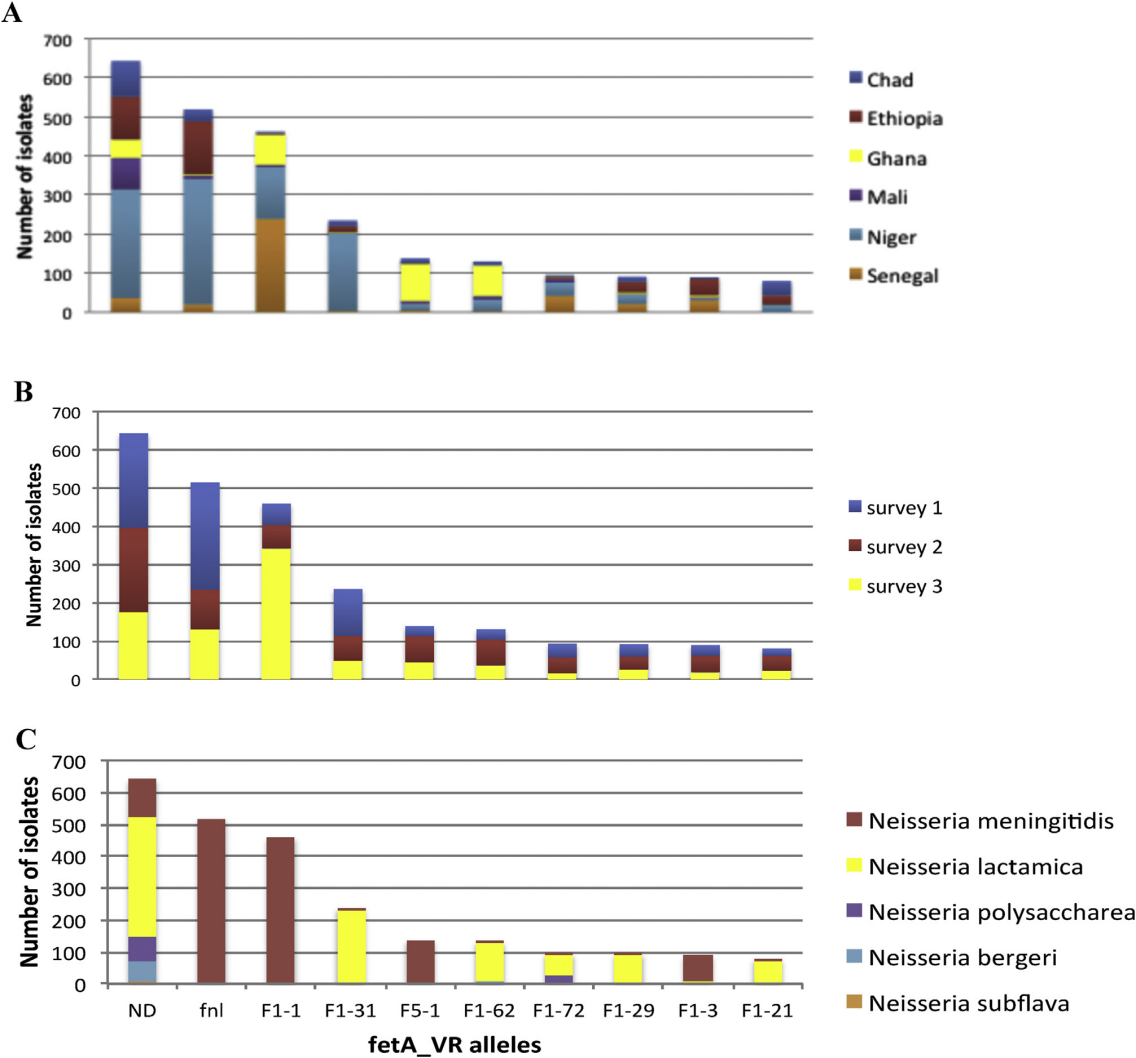
Frequency distribution of *fetA_VR* alleles. Frequency distribution of different alleles of the variable region of *fetA*. Only the 10 most common alleles are displayed; distribution by country (A), by survey (B) and by species (C).

The diversity of *fetA* alleles varied by country (Fig. 3A; Supplementary Table 3); Niger had the highest number of *fetA_vr* alleles (111) and Ghana had the least (55). Some allele variability was seen between surveys, for example the proportion of the F1-1 allele increased from 61 (3.63%) in survey 2 to 342 (23.38%) in survey 3 and similar changes were observed for others alleles in survey 3 (Fig. 3B; Supplementary Table 4). A total of 234 different alleles were identified across all species: 75 of these were found in only one isolate; 184 in fewer than 20 isolates. A total of 194 alleles were observed for *Nl*, 80 for *Nm*; 35 for *Np*; 21 for *Nb*; and 16 for *Ns*. Most alleles were found predominantly in only one species, for example 99.61% of *fnl* alleles were found in *Nm*,^29^, ^30^ all F5-84 variants were only detected in *Nl*, all F5-1 were exclusive to *Nm*, all F11-4 were found only in *Np* and all F1-169 and all F1-193 variants were only observed in *Ns.* Some variants, however, were shared among different species, e.g. F1-72, F1-21, F2-24, F6-3 (Fig. 3C; Supplementary Table 5). Neither the *fetA_VR* nor the *fnl* fragment was successfully amplified in 643 isolates, which were designated Not Determined (ND). As defined by *f_rplf* and *feta_VR* alleles, there were 636 different *Neisseria* strain types identified, with almost half of these (297, 46.70%) observed only once. There was appreciable variation in the frequency of the strain types observed more than 20 times (Table 3).

## Discussion

Although the introduction of PsA-TT into the African meningitis belt has had a major impact on serogroup A epidemics,^8^, ^31^ the region will remain at risk of meningococcal disease until comprehensive vaccines targeting all serogroups are available.^3^ The reasons for the unique epidemiology of the African meningitis belt remain poorly understood,^32^ making it difficult to predict when and where epidemics caused by non-serogroup A meningococci might occur. Variations in the prevalence of NPN species, which potentially contribute cross immunity through sub-capsular antigens, could play a role in determining susceptibility to a potentially epidemic strain. A number of studies have indicated the movements of genes encoding various protein antigens from NPN to *Nm*^14^, ^33^ and variants of the FetA antigen have been previously shown to be shared amongst *Neisseria* species.^14^, ^34^ This antigen is known to generate protective responses in humans.^35^ Colonization with NPNs also affects colonization of humans in experimental studies.^18^

The novel sequence based techniques employed in this study enabled *Nm* and NPN to be identified and characterized rapidly and cost effectively from the very large numbers of samples obtained in the African centers. The *rplF* assay^22^ achieved reliable speciation, which would not have been possible with conventional methods such as 16S rRNA gene sequencing. An indication of diversity within species was achieved by sequencing the variable region of the gene encoding FetA (*fetA_VR*), an outer membrane protein (OMP) found in most *Neisseria*, and which is involved in iron metabolism and been shown to elicit protective immune responses.^36^ Further characterization of the meningococcal isolates has been previously published.^12^

Of the five known *Neisseria* species identified, with a possible novel species present in Chad, the most common were *Nl* and *Nm*. These are the species that have been observed mostly frequently in previous investigations in the African meningitis belt and they are known to have antagonistic interactions in colonization.^18^, ^37^ It is possible that the isolation of some of the other species was affected by the selective media used in this and other studies. Although growth of *Np*,^38^ *Nl*^17^, ^39^, ^40^, ^41^ and *Ns*^42^ on colistin containing agar (such as modified Thayer–Martin or New York city medium) has been reported, other species such as *Neisseria perflava*, *Neisseria sicca*, *Neisseria mucosa, Neisseria cinerea* may not have been identified using the culture technique employed in this study.^43^ The impact of these media on *Nb* is unknown. In future, the identification of NPNs could be enhanced by the use of molecular approaches, although these will have to have species-level resolution, such as the *rplF* assay.^22^ The *Neisseria* identified were highly diverse at both the strain and the species level and varied markedly over time and place. This is consistent with other carriage studies in the meningitis belt, but is different from the relatively stable carriage observed in countries that do not experience large-scale epidemic disease.^44^

The most common NPNs recovered, *Np* and *Nl*, were isolated predominantly from young children as opposed to *Nm*, which was isolated most frequently from older children. *Nl* is known to colonize infants and young children preferentially in many settings^45^ and this is consistent with carriage of NPNs having a role in the rapid acquisition of antibody against *Nm* among children in the meningitis belt, although given the age distribution of meningococcal disease these antibodies may not be protective.^46^

The risk factors for Nm and NPNs carriage were not the same and in some cases (eg. age, sex and season) were inversely related. Individuals were more likely to carry NPNs during the rainy season if they were a female and under 5 years old whereas they were more likely to carry *Nm* during the dry season, if they were male and between 5 and 29 years old.^12^ These results suggest that the physical presence of an NPN in the pharynx may prevent the colonization by *Nm* although this may not apply to the hyper-invasive meningococci since the incidence of meningococcal disease in the African meningitis belt is highest in under five year olds. Recent vaccination with a meningitis vaccine (primarily MenAfriVac^®^, which was used in Mali, Niger and Chad during the course of our study) appeared to be protective both against carriage of Nm and NPNs overall in the regression analysis. This effect could possibly be explained by disturbance of the microbiome following vaccination but could also be due to residual confounding or a reflection of the naturally dynamic patterns of carriage. In Mali, an increase in Nm was observed post-vaccination, due to an expansion of serogroup W.^12^ This seems unlikely to be an effect of MenAfriVac^®^ but it is not known how the removal of one serogroup from the nasopharyngeal environment could affect the other serogroups. The influence of host factors on carriage of *Nm* and NPNs has not been investigated in this study but the IgA secretory status of the individuals sampled could also play a role as previously shown.^47^

Few published studies have characterized pharyngeal NPNs in the African meningitis belt. One recent study in Burkina Faso, which did not survey adults over the age of 30 years or identify species other than *Nm* and *Nl*,^17^ reported a number of differences compared to the MenAfriCar surveys described here: there was a higher overall prevalence of *Nl* carriage (18.2%); a higher prevalence in males, although it was significantly higher in women (9.1% vs 3.9%) for the 18–29 year age group and no significant changes between seasons or post vaccination. The reasons for these inconsistencies are unclear, although both studies indicated fluctuations in prevalence among surveys and these differences may reflect the highly dynamic nature of *Neisseria* carriage.

Some of the *fetA_VR* alleles found were shared among the different species, as reported previously.^14^, ^34^ Since the variable region of *fetA* plays an important role in its immunogenicity,^28^ the shared protein variants could create a cross-reactive immunity at the subscapular level. The inclusion of *fetA*_*VR* in the meningococcal typing system also increased discrimination between strains (supplemental Table 6). Although there was correlation between *porA* and *fetA_VR* alleles for some *Nm* strains (e.g. *cnl*:P1.18-11,42-1:*fnl*), in others the *fetA*_*VR* (e.g. W:P1.5,2:F1-1 and W:P1.5,2:F6-3) or both OMPs (e.g. W:P15-1,2-36:F5-1) sequences varied, suggesting that the serogroup W *Nm* strains are antigenically diverse. This has potential implications for using proteins such as *porA* or *fetA* in vaccine formulations.^48^ The Neighbor Joining phylogeny (Supplemental Fig. 3) shows the clustering of all of the isolates with the appropriate species except for one, which has the *f_rplF* allele 58 defined previously as *Ns*^22^ but clusters more closely, in this study, with the *Nb* species which was not discovered at the time of the previous study. This particular isolate may also be a *Ns* with a *f_rplF* allele similar to *Nb*. More sequence data from this isolate will clarify this issue.

This study has demonstrated the dynamic nature and high diversity of the genus *Neisseria* in pharyngeal carriage in countries of the African meningitis belt. The results are consistent with the idea that the carriage of NPNs may influence invasive meningococcal disease epidemiology in the African meningitis belt. Although more research is needed to elucidate such effects, understanding these organisms could potentially contribute to meningococcal disease control. This is of particular importance given the absence of comprehensive vaccines against all meningococcal serogroups and the continuing interest in the development of protein based vaccines.

## Financial support

MenAfriCar was funded by the Wellcome Trust (086546/Z/08/Z) and the Bill and Melinda Gates Foundation (51251). Kanny Diallo holds a Wellcome Trust Training Fellowship in Public Health and Tropical Medicine. The funding sources had no role in the study design, collection, analysis and interpretation of the data, in the writing of the report or the decision to submit the paper for publication.

## Conflict of interest

Caroline Trotter reports that she received a Consulting payment in 2013 for a critical review of health economic model of meningococcal ACWY vaccine by GlaxoSmithKline (GSK); Ray Borrow reports that he performed contract researches on behalf of Public Health England for Novartis Vaccines and Diagnostics, Baxter Biosciences, Sanofi Pasteur, Serum Institute of India and GSK. All other authors report no potential conflicts of interest.

## Figures and Tables

**Table 1:** Factors associated with carriage of *Neisseria meningitidis* and non-pathogenic *Neisseria*; results from a multinomial multivariable logistic regression.

Factor		Number (carriers)	Adjusted RRR *Neisseria meningitidis* (95% CI)	Adjusted RRR Non-pathogenic *Neisseria* (95% CI)
Age	<1 year	2074 (207)	0.40 (0.29, 0.55)	3.11 (2.60, 3.73)
1–4 years	8291 (1355)	0.70 (0.59, 0.82)	5.90 (5.23, 6.65)
5–14 years	12,563 (895)	1.49 (1.31, 1.69)	2.59 (2.29, 2.94)
15–29 years	11,863 (4372)	1.0	1.0
30 + years	11,243 (206)	0.59 (0.50, 0.68)	0.51 (0.43, 0.61)
Sex^a^	Female	26,619 (1701)	1.0	1.0
Male	19,296 (1313)	1.34 (1.21, 1.48)	0.87 (0.80, 0.94)
Season	Rainy (XS1 and XS2)	30,522 (220)	1.0	1.0
Dry (XS3)	15,512 (815)	1.53 (1.35, 1.74)	0.78 (0.70, 0.86)
Country	Chad	13,396 (584)	1.0	1.0
Ethiopia	5970 (450)	7.11 (5.43, 9.29)	1.70 (1.44, 2.01)
Ghana	5209 (253)	4.81 (3.64, 6.35)	1.25 (1.03, 1.51)
Mali	8837 (219)	1.45 (1.05, 1.99)	0.48 (0.40, 0.58)
Niger	8213 (1112)	11.39 (9.00, 14.43)	3.58 (3.13, 4.01)
Senegal	4409 (427)	10.85 (8.28, 14.23)	2.39 (1.97, 2.89)
Area	Urban	19,462 (1398)	1.0	1.0
Rural	26,572 (1637)	1.44 (1.09, 1.60)	0.97 (0.88, 1.06)
Crowding^b^	<2 people per room	16,299 (889)	1.0	1.0
≥2 people per room	29,679 (2127)	1.27 (1.12, 1.45)	1.08 (0.98, 1.19)
Kitchen location	Open air	17,805 (1274)	1.0	1.0
Inside house	12,741 (1027)	1.32 (1.09, 1.60)	0.90 (0.79, 1.02)
Separate hut	14927 (677)	0.94 (0.76, 1.17)	0.96 (0.84, 1.09)
Missing information	504 (38)	1.06 (0.57, 1.98)	0.77 (0.51, 1.17)
Vaccinated recently with meningitis vaccine	No	31,338 (2060)	1.0	1.0
Yes, <1 year ago	9048 (545)	0.71 (0.59, 0.84)	0.82 (0.73, 0.92)
Yes, 1–3 years ago	4543 (356)	0.51 (0.42, 0.63)	0.94 (0.81, 1.10)
Don't know/missing	1020 (55)	0.68 (0.47, 1.00)	0.62 (0.46, 0.84)

The final multivariable logistic regression model included age group, sex, country, season area, crowding, kitchen location and recent vaccination.

Other risk factors used in the univariable model but not significant in the adjusted model: smoking, living in a house with smokers, cooking fuel, respiratory symptoms and attendance of social gatherings.

a Sex not reported for 119 individuals.

b Data not reported for 57 individuals.

**Table 2:** Number of isolates identified as a *Neisseria* species per survey.

	*N. meningitidis*	Prevalence [CI]	*N. lactamica*	Prevalence [CI]	*N. polysaccharea*	Prevalence [CI]	*N. bergeri*	Prevalence [CI]	*N. subflava*	Prevalence [CI]	Total	Prevalence [CI]
Survey 1	577	3.6 [3.3–3.9]	896	5.7 [5.3–6.1]	79	0.5 [0.4–0.6]	17	0.1 [0.1–0.2]	4	0.03 [0–0.05]	1573	10.0 [9.4–10.5]
Survey 2	454	3.0 [2.7–3.3]	1010	6.7 [6.3–7.2]	117	0.8 [0.62–0.9]	62	0.4 [0.3–0.5]	17	0.1 [0.1–0.2]	1660	11.1 [10.6–11.7]
Survey 3	648	4.2 [3.8–4.5]	682	4.4 [4.0–4.7]	94	0.6 [0.5–0.7]	34	0.2 [0.1–0.3]	3	0.02 [−0.002 to 0.04]	1461	9.41 [8.9–9.9]
**Total**	**1679**	**3.6 [3.4**–**3.8]**	**2588**	**5.6 [5.3**–**5.8]**	**290**	**0.63 [0.5**–**0.7]**	**113**	**0.2 [0.2**–**0.3]**	**24**	**0.05 [0.03**–**0.1]**	**4694**	**10.2 [9.8**–**10.5]**

**Table 3:** Number of isolates for the most common commensal strain types per species.

Commensal strain_type “*f_ rplf:fetA*”	*Neisseria bergeri*	*Neisseria lactamica*	*Neisseria meningitidis*	*Neisseria polysaccharea*	Total
1:fnl	–	–	470	–	470
2:F1-1	–	–	452	–	452
6:F1-31	–	205	–	–	205
2:F5-1	–	–	135	–	135
6:ND	–	122	–	–	122
32:ND	–	88	–	–	88
52:ND	–	82	0	–	82
2:F1-3	–	–	74	–	74
1:ND	–	–	68	–	68
9:ND	–	–	–	66	66
6:F1-29	–	62	–	–	62
34:F1-62	–	56	–	–	56
6:F1-62	–	47	–	–	47
52:F3-60	–	46	–	–	46
6:F1-100	–	46	–	–	46
6:F1-72	–	46	–	–	46
1:F3-1	–	–	45	–	45
6:F5-133	–	45	–	–	45
6:F4-5	–	43	–	–	43
6:F4-17	–	42	–	–	42
9:F7-3	–	–	–	37	37
6:F5-84	–	36	–	–	36
33:F2-17	–	35	–	–	35
52:F1-21	–	35	–	–	35
62:ND	35	–	–	–	35
33:ND	–	34	–	–	34
88:fnl	–	–	32	–	32
9:F2-24	–	–	–	32	32
1:F5-5	–	–	31	–	31
34:ND	–	31	–	–	31
69:ND	31	–	–	–	31
6:F4-6	–	29	–	–	29
6:F5-12	–	29	–	–	29
2:ND	–	–	28	–	28
6:F5-18	–	26	–	–	26
2:F6-3	–	–	25	–	25
52:F1-29	–	25	–	–	25
6:F1-21	–	25	–	–	25
9:F11-4	–	–	–	25	25
4:F1-7	–	–	23	–	23
63:F1-72	–	–	–	23	23
6:F6-2	–	23	–	–	23
1:F4-23	–	–	22	–	22
33:F4-6	–	22	–	–	22
33:F5-34	–	22	–	–	22
6:F2-23	–	22	–	–	22
6:F1-120	–	21	–	–	21
6:F1-101	–	20	–	–	20
