# Supplementary material for: Pharyngeal carriage of Neisseria species in the African meningitis belt
Source: J Infect. 2016 Jun;72(6):667–77. doi: 10.1016/j.jinf.2016.03.010 (PMC4879866; doi:10.1016/j.jinf.2016.03.010)
Supplement: Supplementary file 1 [file mmc1.doc]

**Pharyngeal carriage of *Neisseria* species in the African meningitis belt**

**Supplementary methods**

**General Sanger Sequencing protocol**

A reaction mixture of 50μl was prepared for the PCR amplification step containing 10μl of Q Solution Buffer (Quiagen), 5μl of 10X Buffer (Quiagen), 1μl of dNTPs (Quiagen), 1μl of each primer (10uM stocks), 0.25μl Taq DNA polymerase (Quiagen) and 2μl of DNA, with the following reaction conditions: one cycle at 95 °C for 3 min, followed by 35 cycles of a sequence of three temperatures, 95°C for 30 s, 55°C for 30 s and 72°C for 1 min and a final incubation of one cycle at 72 °C for a further 10 min. The amplified reactions were purified using the PEG-NaCl precipitation method [32]. Sequencing of the cleaned DNA precipitate was done in a 10μl reaction mix containing the following: 0.5μl BigDye ready reaction mix (Life Technologies), 1.75μl of 5X dilution buffer (Life Technologies), 4μl of primer (10μM 1:15) and 2μl of DNA, with cycling condition as follow: 30 cycles of a sequence of tree temperatures, 96°C for 10 s, 50°C for 5 s and 60°C for 2 min. Sequence reactions were cleaned before sequencing on an Applied Biosystems 3730 DNA analyzer (Life Technologies).

***fetA_VR* sequencing protocol**

Primers S1 and S8 [37] were used for the PCR amplification with the following reaction conditions: one cycle at 95 °C for 3 min, followed by 30 cycles of a sequence of three temperatures, 94°C for 1 min, 55°C for 1 min and 72°C for 1 min and a final incubation of one cycle at 72 °C for a further 5 min. The cleaned amplicons were sequenced using primer S12 (forward sequencing) and primer S15 (reverse sequencing) [37].

***fnl* sequencing protocol**

Primers CM21 and CM24 were used for both amplification and sequencing; however, the reaction mixture was slightly different from the general method described above, in that it did not contain the Q Solution Buffer (Quiagen) and the cycling conditions were as follows: one cycle at 95°C for 3 min, followed by 35 cycles of a sequence of three temperatures, 95°C for 30 s, 56°C for 30 s and 72°C for 5 minutes and a final incubation at 72 °C for a further 10 minutes. Sequencing was done as for the other targets

**Supplementary Tables**

**Table S1: Number of isolates identified as a *Neisseria* species per country**

|  | *N meningitidis* | Prevalence  [CI] | *N lactamica* | Prevalence  [CI] | *N polysaccharea* | Prevalence  [CI] | *N bergeri* | Prevalence  [CI] | *N subflava* | Prevalence  [CI] | **Total** | Prevalence  [CI] |
| --- | --- | --- | --- | --- | --- | --- | --- | --- | --- | --- | --- | --- |
| *Chad N=13396 | 131 | 0.9 [0.8-1.1] | 467 | 3.5 [3.1-3.8] | 40 | 0.3 [0.2-0.4] | 47 | 0.3 [0.2-0.5] | 10 | 0.1 [0.03-0.1] | **695** | 5.3 [4.9-5.7] |
| Ethiopia N=5970 | 388 | 6.3 [5.6-7.1] | 268 | 4.4 [3.8-5.0] | 179 | 3.0 [2.5-3.5] | 0 | - | 3 | 0.05 [-0.01-0.1] | **838** | 13.8 [12.8-14.8] |
| Ghana N=5209 | 193 | 3.7 [3.1-4.3] | 217 | 4.1 [3.5-4.7] | 10 | 0.2 [0.1-0.3] | 26 | 0.5 [0.3-0.7] | 0 | - | **446** | 8.5 [7.6-9.4] |
| *Mali N=8837 | 79 | 0.9 [0.7-1.1] | 170 | 1.9 [1.6-2.2] | 9 | 0.1 [0.03-0.2] | 37 | 0.4 [0.3-0.6] | 3 | 0.03 [-0.004-0.] | **298** | 3.4 [2.9-3.9] |
| *Niger N=8213 | 532 | 6.4 [5.8-7.0] | 1094 | 13.3 [12.5-14.1] | 13 | 0.2 [0.1-0.2] | 3 | 0.04 [-0.005-0.01] | 2 | 0.02 [-0.01-0.05] | **1644** | 19.9 [18.9-20.9] |
| Senegal N=4409 | 356 | 8.0 [7.1-9.0] | 372 | 8.4 [7.4-9.3] | 39 | 0.9 [0.6-1.2] | 0 | - | 6 | 0.1 [0.03-0.2] | **773** | 17.5 [16.1418.8] |
| **Total** | **1679** | **3.6 [3.4-3.8]** | **2588** | **5.6 [5.3-5.8]** | **290** | **0.6 [0.5-0.7]** | **113** | **0.2 [0.2-0.3]** | **24** | **0.05 [0.03-0.1]** | **4694** | **10.2 [9.8-10.5]** |

N corresponds to the total number of subject studied; CI: Confidence Interval

*Countries that received the serogroup A conjugate vaccine (MenAfriVac) during the study

**Table S2: Frequency of *f_rplf*** alleles by species

| *f_rplF* | *Neisseria bergeri* | *Neisseria lactamica* | *Neisseria meningitidis* | *Neisseria polysaccharea* | *Neisseria subflava* | Total |
| --- | --- | --- | --- | --- | --- | --- |
| 6 | - | 1453 | - | - | - | 1453 |
| 2 | - | - | 749 | - | - | 749 |
| 1 | - | - | 707 | - | - | 707 |
| 52 | - | 453 | - | - | - | 453 |
| 33 | - | 318 | - | - | - | 318 |
| 9 | - | - | - | 207 | - | 207 |
| 32 | - | 186 | - | - | - | 186 |
| 34 | - | 132 | - | - | - | 132 |
| 67 | - | - | 83 | - | - | 83 |
| 63 | - | - | - | 72 | - | 72 |
| 4 | - | - | 62 | - | - | 62 |
| 62 | 57 | - | - | - | - | 57 |
| 69 | 46 | - | - | - | - | 46 |
| 88 | - | - | 41 | - | - | 41 |
| 3 | - | - | 15 | - | - | 15 |
| 43 | - | - | - | - | 15 | 15 |
| 8 | - | - | 10 | - | - | 10 |
| 112 | 9 | - | - | - | - | 9 |
| 51 | - | 8 | - | - | - | 8 |
| 80 | - | 7 | - | - | - | 7 |
| 86 | - | 6 | - | - | - | 6 |
| 90 | - | - | - | 6 | - | 6 |
| 18 | - | - | 5 | - | - | 5 |
| 65 | - | 5 | - | - | - | 5 |
| 81 | - | 5 | - | - | - | 5 |
| 96 | - | - | - | 5 | - | 5 |
| 113 | - | 4 | - | - | - | 4 |
| 64 | - | - | 3 | - | - | 3 |
| 82 | - | - | - | - | 3 | 3 |
| 91 | - | - | 3 | - | - | 3 |
| 92 | - | 3 | - | - | - | 3 |
| 94 | - | 3 | - | - | - | 3 |
| 97 | - | - | - | - | 2 | 2 |
| 111 | - | 2 | - | - | - | 2 |
| 0 | - | - | 1 | - | - | 1 |
| 16 | 1 | - | - | - | - | 1 |
| 31 | - | - | - | - | 1 | 1 |
| 38 | - | - | - | - | 1 | 1 |
| 58 | - | - | - | - | 1 | 1 |
| 85 | - | 1 | - | - | - | 1 |
| 89 | - | 1 | - | - | - | 1 |
| 93 | - | 1 | - | - | - | 1 |
| 95 | - | - | - | - | 1 | 1 |
| Total | 113 | 2588 | 1679 | 290 | 24 | 4694 |

**TableS3: Frequency of *feta_VR*** alleles by countries

| *fetA_VR* | Chad | Ethiopia | Ghana | Mali | Niger | Senegal | Total |
| --- | --- | --- | --- | --- | --- | --- | --- |
| ND | 92 | 109 | 48 | 80 | 278 | 36 | 643 |
| *fnl* | 31 | 135 | 2 | 10 | 321 | 20 | 519 |
| F1-1 | 2 | 4 | 77 | 6 | 132 | 239 | 460 |
| F1-31 | 15 | 15 | 2 | 1 | 199 | 3 | 235 |
| F5-1 | 13 | 1 | 95 | 8 | 17 | 4 | 138 |
| F1-62 | 10 | - | 79 | 9 | 30 | 2 | 130 |
| F1-72 | 2 | 5 | 1 | 9 | 34 | 42 | 93 |
| F1-29 | 14 | 26 | 3 | 2 | 25 | 21 | 91 |
| F1-3 | 4 | 42 | 3 | 6 | 4 | 30 | 89 |
| F1-21 | 37 | 24 | - | - | 19 | - | 80 |
| F4-5 | 7 | 4 | 13 | 3 | 23 | 16 | 66 |
| F4-6 | 15 | 1 | 1 | 4 | 32 | 12 | 65 |
| F1-100 | 3 | - | 7 | 1 | 16 | 32 | 59 |
| F5-133 | 6 | 3 | - | 2 | 2 | 44 | 57 |
| F2-24 | 2 | 42 | 4 | 2 | 1 | 5 | 56 |
| F5-34 | 20 | 14 | 1 | 6 | 10 | 3 | 54 |
| F3-1 | 44 | 1 | - | - | - | 7 | 52 |
| F6-3 | 1 | - | - | 30 | 5 | 16 | 52 |
| F7-3 | 2 | 44 | - | - | 3 | - | 49 |
| F3-60 | - | 47 | - | - | - | - | 47 |
| F6-2 | 12 | 3 | 1 | 2 | 20 | 8 | 46 |
| F4-17 | 14 | - | 9 | - | 19 | 3 | 45 |
| F1-24 | 8 | 11 | - | - | 25 | - | 44 |
| F5-84 | 4 | - | 2 | 22 | 2 | 13 | 43 |
| F1-120 | 14 | - | 1 | 3 | 22 | 1 | 41 |
| F5-12 | 8 | 2 | 3 | 9 | 13 | 3 | 38 |
| F2-17 | - | - | - | 1 | 34 | 2 | 37 |
| F3-22 | 5 | 10 | - | 4 | 14 | 2 | 35 |
| F1-155 | 10 | - | 4 | 17 | - | 3 | 34 |
| F5-5 | 1 | 27 | 2 | - | - | 4 | 34 |
| F2-23 | 3 | - | 6 | 1 | 20 | 3 | 33 |
| F1-101 | 16 | - | 3 | 2 | 11 | - | 32 |
| F1-153 | 12 | 17 | - | - | 1 | 1 | 31 |
| F1-160 | 15 | - | 1 | 1 | 11 | 2 | 30 |
| F4-19 | 21 | 4 | - | - | 4 | - | 29 |
| F1-7 | - | 20 | 1 | - | 3 | 4 | 28 |
| F5-66 | 14 | - | 6 | 1 | 5 | 2 | 28 |
| F1-145 | 9 | 9 | 1 | 2 | 6 | - | 27 |
| F1-38 | - | - | - | 3 | 1 | 23 | 27 |
| F5-18 | 10 | - | 9 | - | 8 | - | 27 |
| F4-23 | 14 | 9 | 2 | - | 1 | - | 26 |
| F5-88 | 6 | - | 1 | - | 13 | 6 | 26 |
| F1-104 | 3 | - | 11 | 2 | - | 9 | 25 |
| F1-58 | - | - | - | 6 | 3 | 16 | 25 |
| F1-84 | 2 | 11 | - | 1 | 11 | - | 25 |
| F11-4 | - | 25 | - | - | - | - | 25 |
| F1-156 | 5 | - | 2 | 4 | 6 | 4 | 21 |
| F1-46 | - | - | - | - | 21 | - | 21 |
| F1-49 | 7 | 6 | - | - | 6 | 2 | 21 |
| F3-57 | - | - | - | 2 | 17 | 1 | 20 |
| F4-28 | 3 | 2 | 1 | - | 11 | 3 | 20 |
| F1-186 | 3 | 10 | - | - | 6 | - | 19 |
| F5-127 | 2 | 4 | 1 | 1 | 5 | 6 | 19 |
| F4-2 | - | - | - | 1 | - | 17 | 18 |
| F5-25 | 1 | - | - | - | 17 | - | 18 |
| F3-3 | 6 | 6 | 1 | 3 | - | 1 | 17 |
| F5-69 | 4 | 1 | 6 | - | 5 | 1 | 17 |
| F5-8 | - | 14 | 2 | - | - | - | 16 |
| F6-7 | - | 16 | - | - | - | - | 16 |
| F1-173 | 10 | - | - | - | 4 | - | 14 |
| F1-35 | - | 8 | - | - | 6 | - | 14 |
| F1-81 | - | - | - | - | - | 14 | 14 |
| F3-56 | - | - | 2 | - | 12 | - | 14 |
| F4-1 | 1 | 7 | - | 1 | - | 5 | 14 |
| F1-89 | 1 | - | - | - | 10 | 1 | 12 |
| F3-43 | 4 | 4 | - | 1 | 1 | 2 | 12 |
| F5-101 | - | - | - | 1 | 8 | 2 | 11 |
| F5-140 | 6 | - | 1 | - | 4 | - | 11 |
| F6-6 | 1 | 4 | - | - | 6 | - | 11 |
| F1-28 | - | 8 | 1 | - | 1 | - | 10 |
| F3-18 | - | 9 | - | 1 | - | - | 10 |
| F5-77 | 1 | - | - | - | 8 | 1 | 10 |
| F1-197 | - | - | - | - | 6 | 3 | 9 |
| F4-51 | 2 | - | - | 2 | 5 | - | 9 |
| F5-24 | - | - | - | 8 | 1 | - | 9 |
| F1-22 | 8 | - | - | - | - | - | 8 |
| F3-12 | 2 | 1 | - | - | - | 5 | 8 |
| F5-149 | - | - | 4 | - | 4 | - | 8 |
| F5-9 | 1 | 5 | 2 | - | - | - | 8 |
| F1-174 | 7 | - | - | - | - | - | 7 |
| F1-192 | - | - | - | - | 6 | 1 | 7 |
| F2-14 | - | 6 | - | - | - | 1 | 7 |
| F2-6 | - | - | - | - | - | 7 | 7 |
| F4-38 | 5 | - | 1 | - | 1 | - | 7 |
| F5-70 | 4 | - | 1 | - | 2 | - | 7 |
| F5-87 | 1 | - | - | - | 6 | - | 7 |
| F1-170 | 6 | - | - | - | - | - | 6 |
| F1-172 | 4 | - | - | - | 2 | - | 6 |
| F1-5 | 2 | 2 | 1 | - | - | 1 | 6 |
| F2-32 | - | - | - | - | - | 6 | 6 |
| F7-2 | 5 | - | - | - | 1 | - | 6 |
| F1-183 | - | - | 2 | - | 2 | 1 | 5 |
| F1-20 | - | 5 | - | - | - | - | 5 |
| F1-209 | - | - | 4 | - | 1 | - | 5 |
| F1-55 | - | 5 | - | - | - | - | 5 |
| F3-28 | - | - | - | - | 4 | 1 | 5 |
| F5-132 | 3 | - | - | 1 | 1 | - | 5 |
| F5-17 | 1 | 2 | - | - | - | 2 | 5 |
| F5-71 | - | - | - | 3 | 1 | 1 | 5 |
| F6-4 | - | - | - | 1 | - | 4 | 5 |
| F1-152 | 4 | - | - | - | - | - | 4 |
| F1-177 | 4 | - | - | - | - | - | 4 |
| F1-189 | - | - | 4 | - | - | - | 4 |
| F1-47 | - | 1 | - | - | 3 | - | 4 |
| F1-51 | - | 4 | - | - | - | - | 4 |
| F1-80 | - | 1 | - | - | - | 3 | 4 |
| F1-96 | 4 | - | - | - | - | - | 4 |
| F11-3 | 1 | - | 3 | - | - | - | 4 |
| F2-9 | - | 4 | - | - | - | - | 4 |
| F5-137 | - | - | - | - | - | 4 | 4 |
| F5-145 | 4 | - | - | - | - | - | 4 |
| F5-4 | - | 1 | - | - | 2 | 1 | 4 |
| F5-59 | - | 4 | - | - | - | - | 4 |
| F5-91 | 2 | 2 | - | - | - | - | 4 |
| F1-176 | 1 | - | - | - | 2 | - | 3 |
| F1-181 | 3 | - | - | - | - | - | 3 |
| F1-182 | 3 | - | - | - | - | - | 3 |
| F1-184 | - | 3 | - | - | - | - | 3 |
| F1-187 | - | 1 | - | - | 2 | - | 3 |
| F1-196 | - | - | - | - | 2 | 1 | 3 |
| F1-37 | - | - | - | 1 | - | 2 | 3 |
| F1-52 | - | - | - | - | - | 3 | 3 |
| F11-1 | 1 | - | - | 2 | - | - | 3 |
| F11-7 | 2 | - | - | - | 1 | - | 3 |
| F2-31 | - | 3 | - | - | - | - | 3 |
| F3-6 | - | - | - | - | - | 3 | 3 |
| F3-9 | 1 | 2 | - | - | - | - | 3 |
| F5-134 | - | 3 | - | - | - | - | 3 |
| F5-142 | 1 | - | - | - | 2 | - | 3 |
| F5-146 | 3 | - | - | - | - | - | 3 |
| F5-95 | - | - | - | 1 | - | 2 | 3 |
| F1-158 | - | - | - | 2 | - | - | 2 |
| F1-175 | 2 | - | - | - | - | - | 2 |
| F1-178 | 2 | - | - | - | - | - | 2 |
| F1-18 | - | 2 | - | - | - | - | 2 |
| F1-190 | - | - | 2 | - | - | - | 2 |
| F1-199 | - | - | - | - | 2 | - | 2 |
| F1-2 | 2 | - | - | - | - | - | 2 |
| F1-200 | - | - | - | - | 2 | - | 2 |
| F1-64 | - | 2 | - | - | - | - | 2 |
| F1-90 | - | - | - | 2 | - | - | 2 |
| F11-2 | - | - | 1 | - | 1 | - | 2 |
| F11-5 | - | - | - | - | 1 | 1 | 2 |
| F11-8 | 2 | - | - | - | - | - | 2 |
| F2-12 | 2 | - | - | - | - | - | 2 |
| F2-13 | 2 | - | - | - | - | - | 2 |
| F3-13 | - | 2 | - | - | - | - | 2 |
| F3-59 | 2 | - | - | - | - | - | 2 |
| F4-57 | - | - | 2 | - | - | - | 2 |
| F4-60 | - | 2 | - | - | - | - | 2 |
| F4-63 | - | - | - | - | 2 | - | 2 |
| F4-64 | - | - | - | - | 2 | - | 2 |
| F4-65 | - | - | - | - | 2 | - | 2 |
| F4-66 | - | - | - | - | 2 | - | 2 |
| F5-143 | 2 | - | - | - | - | - | 2 |
| F5-151 | - | 2 | - | - | - | - | 2 |
| F5-154 | - | - | - | - | - | 2 | 2 |
| F5-155 | - | - | - | - | - | 2 | 2 |
| F5-160 | - | - | - | - | 2 | - | 2 |
| F5-19 | - | - | - | - | - | 2 | 2 |
| F1-119 | - | - | - | - | - | 1 | 1 |
| F1-15 | 1 | - | - | - | - | - | 1 |
| F1-157 | - | - | - | 1 | - | - | 1 |
| F1-159 | - | - | - | 1 | - | - | 1 |
| F1-167 | - | - | - | 1 | - | - | 1 |
| F1-169 | 1 | - | - | - | - | - | 1 |
| F1-171 | 1 | - | - | - | - | - | 1 |
| F1-179 | 1 | - | - | - | - | - | 1 |
| F1-180 | 1 | - | - | - | - | - | 1 |
| F1-185 | - | 1 | - | - | - | - | 1 |
| F1-188 | - | 1 | - | - | - | - | 1 |
| F1-19 | - | - | 1 | - | - | - | 1 |
| F1-191 | - | - | - | - | - | 1 | 1 |
| F1-193 | - | - | - | - | - | 1 | 1 |
| F1-194 | - | - | - | - | - | 1 | 1 |
| F1-195 | - | - | - | - | - | 1 | 1 |
| F1-198 | - | - | - | - | 1 | - | 1 |
| F1-201 | - | - | - | - | 1 | - | 1 |
| F1-202 | - | - | - | - | 1 | - | 1 |
| F1-203 | - | - | - | - | 1 | - | 1 |
| F1-204 | - | - | - | - | 1 | - | 1 |
| F1-205 | - | - | - | - | 1 | - | 1 |
| F1-206 | - | - | - | - | 1 | - | 1 |
| F1-207 | - | - | - | - | 1 | - | 1 |
| F1-208 | - | - | - | - | 1 | - | 1 |
| F1-33 | - | - | - | - | - | 1 | 1 |
| F1-68 | - | 1 | - | - | - | - | 1 |
| F1-94 | - | - | - | - | - | 1 | 1 |
| F1-99 | - | 1 | - | - | - | - | 1 |
| F10-1 | 1 | - | - | - | - | - | 1 |
| F11-6 | - | - | - | - | 1 | - | 1 |
| F2-33 | - | - | - | - | 1 | - | 1 |
| F2-7 | - | - | - | - | 1 | - | 1 |
| F3-5 | - | 1 | - | - | - | - | 1 |
| F3-61 | - | 1 | - | - | - | - | 1 |
| F3-62 | - | 1 | - | - | - | - | 1 |
| F3-63 | - | - | - | - | - | 1 | 1 |
| F3-64 | - | - | - | - | - | 1 | 1 |
| F3-65 | - | - | - | - | 1 | - | 1 |
| F3-66 | - | 1 | - | - | - | - | 1 |
| F4-12 | - | - | - | - | 1 | - | 1 |
| F4-21 | - | - | - | - | - | 1 | 1 |
| F4-24 | 1 | - | - | - | - | - | 1 |
| F4-25 | - | - | - | - | - | 1 | 1 |
| F4-58 | - | - | 1 | - | - | - | 1 |
| F4-59 | - | 1 | - | - | - | - | 1 |
| F4-61 | - | - | - | - | - | 1 | 1 |
| F4-62 | - | - | - | - | - | 1 | 1 |
| F5-115 | - | - | - | - | 1 | - | 1 |
| F5-118 | 1 | - | - | - | - | - | 1 |
| F5-13 | - | - | - | - | - | 1 | 1 |
| F5-131 | - | - | - | 1 | - | - | 1 |
| F5-139 | 1 | - | - | - | - | - | 1 |
| F5-141 | 1 | - | - | - | - | - | 1 |
| F5-144 | 1 | - | - | - | - | - | 1 |
| F5-147 | 1 | - | - | - | - | - | 1 |
| F5-150 | - | 1 | - | - | - | - | 1 |
| F5-152 | - | - | 1 | - | - | - | 1 |
| F5-153 | - | - | - | - | - | 1 | 1 |
| F5-156 | - | - | - | - | - | 1 | 1 |
| F5-157 | - | - | - | - | 1 | - | 1 |
| F5-158 | - | - | - | - | 1 | - | 1 |
| F5-159 | - | - | - | - | 1 | - | 1 |
| F5-161 | 1 | - | - | - | - | - | 1 |
| F5-162 | - | - | - | - | 1 | - | 1 |
| F5-163 | - | - | - | - | 1 | - | 1 |
| F5-164 | 1 | - | - | - | - | - | 1 |
| F5-165 | 1 | - | - | - | - | - | 1 |
| F5-166 | 1 | - | - | - | - | - | 1 |
| F5-2 | - | - | - | - | - | 1 | 1 |
| F5-67 | 1 | - | - | - | - | - | 1 |
| F5-68 | - | 1 | - | - | - | - | 1 |
| F5-81 | - | 1 | - | - | - | - | 1 |
| F6-5 | - | - | - | 1 | - | - | 1 |
| F7-4 | 1 | - | - | - | - | - | 1 |
| Total | 715 | 841 | 446 | 299 | 1645 | 773 | 4699 |

**Table S4: Frequency of *fetA_VR* alleles by survey**

| *fetA_VR* | survey 1 | survey 2 | survey 3 | Total |
| --- | --- | --- | --- | --- |
| ND | 247 | 221 | 175 | 643 |
| *fnl* | 280 | 104 | 130 | 514 |
| F1-1 | 56 | 61 | 342 | 459 |
| F1-31 | 122 | 65 | 48 | 235 |
| F5-1 | 26 | 68 | 44 | 138 |
| F1-62 | 27 | 67 | 36 | 130 |
| F1-72 | 36 | 42 | 15 | 93 |
| F1-29 | 32 | 34 | 25 | 91 |
| F1-3 | 28 | 43 | 18 | 89 |
| F1-21 | 19 | 39 | 22 | 80 |
| F4-5 | 25 | 22 | 19 | 66 |
| F4-6 | 30 | 18 | 17 | 65 |
| F1-100 | 16 | 25 | 18 | 59 |
| F5-133 | 19 | 22 | 16 | 57 |
| F2-24 | 9 | 27 | 19 | 55 |
| F5-34 | 15 | 27 | 12 | 54 |
| F3-1 | 10 | 40 | 2 | 52 |
| F6-3 | 9 | 14 | 29 | 52 |
| F7-3 | 15 | 13 | 20 | 48 |
| F3-60 | 6 | 24 | 17 | 47 |
| F6-2 | 18 | 12 | 16 | 46 |
| F4-17 | 11 | 17 | 17 | 45 |
| F1-24 | 9 | 23 | 12 | 44 |
| F5-84 | 22 | 9 | 12 | 43 |
| F1-120 | 16 | 17 | 8 | 41 |
| F2-17 | 26 | 4 | 7 | 37 |
| F5-12 | 20 | 9 | 8 | 37 |
| F3-22 | 18 | 9 | 8 | 35 |
| F1-155 | 15 | 14 | 5 | 34 |
| F5-5 | 16 | 11 | 7 | 34 |
| F2-23 | 12 | 15 | 6 | 33 |
| F1-101 | 5 | 18 | 9 | 32 |
| F1-153 | 4 | 17 | 10 | 31 |
| F1-160 | 5 | 11 | 14 | 30 |
| F4-19 | 2 | 23 | 4 | 29 |
| F1-7 | 12 | 9 | 7 | 28 |
| F5-66 | 4 | 15 | 9 | 28 |
| F1-145 | 6 | 13 | 8 | 27 |
| F1-38 | 8 | 16 | 3 | 27 |
| F5-18 | 3 | 13 | 11 | 27 |
| F4-23 | 7 | 10 | 9 | 26 |
| F5-88 | 7 | 5 | 14 | 26 |
| F1-104 | 2 | 11 | 12 | 25 |
| F1-58 | 11 | 10 | 4 | 25 |
| F1-84 | 8 | 5 | 12 | 25 |
| F11-4 | 12 | 7 | 6 | 25 |
| F1-156 | 7 | 12 | 2 | 21 |
| F1-46 | 11 | 2 | 8 | 21 |
| F1-49 | 7 | 8 | 6 | 21 |
| F3-57 | 10 | 9 | 1 | 20 |
| F4-28 | 8 | 9 | 3 | 20 |
| F5-127 | 8 | 7 | 4 | 19 |
| F1-186 | 5 | 8 | 5 | 18 |
| F4-2 | 5 | 13 | - | 18 |
| F5-25 | 13 | 5 | - | 18 |
| F3-3 | 4 | 11 | 2 | 17 |
| F5-69 | 2 | 13 | 2 | 17 |
| F5-8 | 12 | 4 | - | 16 |
| F6-7 | 7 | 5 | 4 | 16 |
| F1-173 | 1 | 9 | 4 | 14 |
| F1-35 | 7 | 3 | 4 | 14 |
| F1-81 | 4 | 8 | 2 | 14 |
| F3-56 | 5 | 4 | 5 | 14 |
| F4-1 | 6 | 5 | 3 | 14 |
| F1-89 | 9 | 3 | - | 12 |
| F3-43 | 6 | 6 | - | 12 |
| F5-101 | 6 | 4 | 1 | 11 |
| F5-140 | 3 | 5 | 3 | 11 |
| F6-6 | 1 | 4 | 6 | 11 |
| F1-28 | - | 8 | 2 | 10 |
| F3-18 | 3 | 5 | 2 | 10 |
| F5-77 | 3 | 3 | 4 | 10 |
| F1-197 | 6 | 1 | 2 | 9 |
| F4-51 | 4 | 4 | 1 | 9 |
| F5-24 | 5 | 4 | - | 9 |
| F1-22 | 1 | 6 | 1 | 8 |
| F3-12 | 1 | 5 | 2 | 8 |
| F5-149 | 3 | 4 | 1 | 8 |
| F5-9 | 2 | 4 | 2 | 8 |
| F1-174 | - | 3 | 4 | 7 |
| F1-192 | 5 | - | 2 | 7 |
| F2-14 | 1 | 2 | 4 | 7 |
| F2-6 | 1 | 4 | 2 | 7 |
| F4-38 | - | 5 | 2 | 7 |
| F5-70 | - | 6 | 1 | 7 |
| F5-87 | 3 | 3 | 1 | 7 |
| F1-170 | 1 | 5 | - | 6 |
| F1-172 | 2 | 3 | 1 | 6 |
| F1-5 | - | 4 | 2 | 6 |
| F2-32 | 3 | 2 | 1 | 6 |
| F7-2 | - | 4 | 2 | 6 |
| F1-183 | 2 | 2 | 1 | 5 |
| F1-20 | - | 3 | 2 | 5 |
| F1-209 | 1 | - | 4 | 5 |
| F1-55 | 3 | 2 | - | 5 |
| F3-28 | 2 | - | 3 | 5 |
| F5-132 | - | 4 | 1 | 5 |
| F5-17 | 2 | 2 | 1 | 5 |
| F5-71 | 2 | 2 | 1 | 5 |
| F6-4 | 2 | - | 3 | 5 |
| F1-152 | - | 1 | 3 | 4 |
| F1-177 | - | 2 | 2 | 4 |
| F1-189 | - | 1 | 3 | 4 |
| F1-47 | - | 1 | 3 | 4 |
| F1-51 | - | 3 | 1 | 4 |
| F1-80 | 2 | 2 | - | 4 |
| F1-96 | - | 2 | 2 | 4 |
| F11-3 | - | 4 | - | 4 |
| F2-9 | 2 | 2 | - | 4 |
| F5-137 | - | 1 | 3 | 4 |
| F5-145 | - | 2 | 2 | 4 |
| F5-4 | 2 | 1 | 1 | 4 |
| F5-59 | - | - | 4 | 4 |
| F5-91 | - | 2 | 2 | 4 |
| F1-176 | 1 | 2 | - | 3 |
| F1-181 | - | 3 | - | 3 |
| F1-182 | - | 3 | - | 3 |
| F1-184 | 1 | - | 2 | 3 |
| F1-187 | 2 | 1 | - | 3 |
| F1-196 | - | 1 | 2 | 3 |
| F1-37 | 1 | 2 | - | 3 |
| F1-52 | - | 3 | - | 3 |
| F11-1 | 1 | 1 | 1 | 3 |
| F11-7 | - | 2 | 1 | 3 |
| F2-31 | - | 2 | 1 | 3 |
| F3-6 | 3 | - | - | 3 |
| F3-9 | 1 | 1 | 1 | 3 |
| F5-134 | 1 | 1 | 1 | 3 |
| F5-142 | - | 2 | 1 | 3 |
| F5-146 | - | 2 | 1 | 3 |
| F5-95 | - | - | 3 | 3 |
| F1-158 | - | 2 | - | 2 |
| F1-175 | - | 2 | - | 2 |
| F1-178 | - | 1 | 1 | 2 |
| F1-18 | 2 | - | - | 2 |
| F1-190 | - | - | 2 | 2 |
| F1-199 | 2 | - | - | 2 |
| F1-2 | 1 | 1 | - | 2 |
| F1-200 | 1 | - | 1 | 2 |
| F1-64 | 1 | 1 | - | 2 |
| F1-90 | - | 1 | 1 | 2 |
| F11-2 | - | - | 2 | 2 |
| F11-5 | - | 1 | 1 | 2 |
| F11-8 | - | 2 | - | 2 |
| F2-12 | - | 1 | 1 | 2 |
| F2-13 | - | - | 2 | 2 |
| F3-13 | 2 | - | - | 2 |
| F3-59 | - | 2 | - | 2 |
| F4-57 | - | 2 | - | 2 |
| F4-60 | - | 1 | 1 | 2 |
| F4-63 | 2 | - | - | 2 |
| F4-64 | 2 | - | - | 2 |
| F4-65 | 1 | 1 | - | 2 |
| F4-66 | - | 2 | - | 2 |
| F5-143 | - | 1 | 1 | 2 |
| F5-151 | - | - | 2 | 2 |
| F5-154 | - | 2 | - | 2 |
| F5-155 | - | 1 | 1 | 2 |
| F5-160 | 1 | - | 1 | 2 |
| F5-19 | - | 2 | - | 2 |
| F1-119 | - | 1 | - | 1 |
| F1-15 | 1 | - | - | 1 |
| F1-157 | - | 1 | - | 1 |
| F1-159 | 1 | - | - | 1 |
| F1-167 | 1 | - | - | 1 |
| F1-169 | 1 | - | - | 1 |
| F1-171 | - | 1 | - | 1 |
| F1-179 | - | - | 1 | 1 |
| F1-180 | - | - | 1 | 1 |
| F1-185 | - | 1 | - | 1 |
| F1-188 | - | - | 1 | 1 |
| F1-19 | - | - | 1 | 1 |
| F1-191 | 1 | - | - | 1 |
| F1-193 | 1 | - | - | 1 |
| F1-194 | 1 | - | - | 1 |
| F1-195 | 1 | - | - | 1 |
| F1-198 | 1 | - | - | 1 |
| F1-201 | 1 | - | - | 1 |
| F1-202 | 1 | - | - | 1 |
| F1-203 | 1 | - | - | 1 |
| F1-204 | 1 | - | - | 1 |
| F1-205 | - | - | 1 | 1 |
| F1-206 | - | - | 1 | 1 |
| F1-207 | 1 | - | - | 1 |
| F1-208 | 1 | - | - | 1 |
| F1-33 | 1 | - | - | 1 |
| F1-68 | - | - | 1 | 1 |
| F1-94 | - | - | 1 | 1 |
| F1-99 | - | - | 1 | 1 |
| F10-1 | - | - | 1 | 1 |
| F11-6 | - | 1 | - | 1 |
| F2-33 | - | 1 | - | 1 |
| F2-7 | 1 | - | - | 1 |
| F3-5 | - | 1 | - | 1 |
| F3-61 | - | 1 | - | 1 |
| F3-62 | - | - | 1 | 1 |
| F3-63 | - | 1 | - | 1 |
| F3-64 | - | - | 1 | 1 |
| F3-65 | 1 | - | - | 1 |
| F3-66 | - | - | - | 1 |
| F4-12 | 1 | - | - | 1 |
| F4-21 | - | 1 | - | 1 |
| F4-24 | - | - | 1 | 1 |
| F4-25 | 1 | - | - | 1 |
| F4-58 | - | - | 1 | 1 |
| F4-59 | 1 | - | - | 1 |
| F4-61 | - | - | - | 1 |
| F4-62 | - | - | 1 | 1 |
| F5-115 | 1 | - | - | 1 |
| F5-118 | - | 1 | - | 1 |
| F5-13 | - | 1 | - | 1 |
| F5-131 | - | 1 | - | 1 |
| F5-139 | - | 1 | - | 1 |
| F5-141 | - | 1 | - | 1 |
| F5-144 | - | 1 | - | 1 |
| F5-147 | - | - | 1 | 1 |
| F5-150 | - | 1 | - | 1 |
| F5-152 | - | 1 | - | 1 |
| F5-153 | 1 | - | - | 1 |
| F5-156 | - | - | 1 | 1 |
| F5-157 | 1 | - | - | 1 |
| F5-158 | 1 | - | - | 1 |
| F5-159 | - | 1 | - | 1 |
| F5-161 | 1 | - | - | 1 |
| F5-162 | 1 | - | - | 1 |
| F5-163 | 1 | - | - | 1 |
| F5-164 | - | 1 | - | 1 |
| F5-165 | - | 1 | - | 1 |
| F5-166 | - | - | 1 | 1 |
| F5-2 | 1 | - | - | 1 |
| F5-67 | - | 1 | - | 1 |
| F5-68 | - | 1 | - | 1 |
| F5-81 | - | - | 1 | 1 |
| F6-5 | - | 1 | - | 1 |
| F7-4 | - | - | 1 | 1 |
| *fnl*;F1-1 | 1 | - | - | 1 |
| *fnl*;F1-186 | - | 1 | - | 1 |
| *fnl*;F2-24 | - | 1 | - | 1 |
| *fnl*;F5-12 | - | - | 1 | 1 |
| *fnl*;F7-3 | - | 1 | - | 1 |
| Total | 1,573 | 1,660 | 1,461 | 4,694 |

**Table S5: Frequency of *fetA_VR* alleles by species**

| *fetA_VR allele* | *Neisseria bergeri* | *Neisseria lactamica* | *Neisseria meningitidis* | *Neisseria polysaccharea* | *Neisseria subflava* | Total |
| --- | --- | --- | --- | --- | --- | --- |
| ND | 67 | 375 | 118 | 75 | 7 | 642 |
| *fnl* | - | 2 | 517 | - | - | 519 |
| F1-1 | - | 1 | 459 | - | - | 460 |
| F1-31 | 1 | 229 | 3 | - | 2 | 235 |
| F5-1 | - | - | 138 | - | - | 138 |
| F1-62 | 4 | 122 | 2 | 1 | 1 | 130 |
| F1-72 | - | 63 | 3 | 27 | - | 93 |
| F1-29 | - | 90 | 1 | - | - | 91 |
| F1-3 | - | 7 | 81 | 1 | - | 89 |
| F1-21 | - | 66 | 11 | 2 | 1 | 80 |
| F4-5 | 2 | 59 | 5 | - | - | 66 |
| F4-6 | - | 56 | 3 | 5 | 1 | 65 |
| F1-100 | - | 58 | - | - | 1 | 59 |
| F5-133 | - | 54 | 3 | - | - | 57 |
| F2-24 | 1 | 13 | 10 | 32 | - | 56 |
| F5-34 | - | 50 | 3 | 1 | - | 54 |
| F3-1 | - | - | 52 | - | - | 52 |
| F6-3 | - | 25 | 25 | 2 | - | 52 |
| F7-3 | - | 3 | 2 | 44 | - | 49 |
| F3-60 | - | 46 | 1 | - | - | 47 |
| F6-2 | 3 | 36 | 1 | 5 | 1 | 46 |
| F4-17 | - | 44 | 1 | - | - | 45 |
| F1-24 | - | 43 | 1 | - | - | 44 |
| F5-84 | - | 43 | - | - | - | 43 |
| F1-120 | - | 40 | - | - | 1 | 41 |
| F5-12 | - | 35 | 3 | - | - | 38 |
| F2-17 | - | 37 | - | - | - | 37 |
| F3-22 | 1 | 24 | 10 | - | - | 35 |
| F1-155 | - | 34 | - | - | - | 34 |
| F5-5 | - | - | 34 | - | - | 34 |
| F2-23 | 1 | 30 | 2 | - | - | 33 |
| F1-101 | 4 | 28 | - | - | - | 32 |
| F1-153 | - | 20 | 6 | 5 | - | 31 |
| F1-160 | 1 | 28 | - | - | 1 | 30 |
| F4-19 | - | 28 | - | - | 1 | 29 |
| F5-66 | - | 16 | 12 | - | - | 28 |
| F1-7 | - | 1 | 27 | - | - | 28 |
| F5-18 | - | 27 | - | - | - | 27 |
| F1-38 | - | 25 | 2 | - | - | 27 |
| F1-145 | 2 | 18 | - | 7 | - | 27 |
| F5-88 | 1 | 25 | - | - | - | 26 |
| F4-23 | - | 2 | 24 | - | - | 26 |
| F1-84 | - | 22 | 2 | - | 1 | 25 |
| F1-58 | 2 | 20 | - | 3 | - | 25 |
| F1-104 | 7 | 16 | 1 | 1 | - | 25 |
| F11-4 | - | - | - | 25 | - | 25 |
| F1-46 | - | 21 | - | - | - | 21 |
| F1-49 | - | 19 | 2 | - | - | 21 |
| F1-156 | - | 16 | 2 | 3 | - | 21 |
| F3-57 | - | 20 | - | - | - | 20 |
| F4-28 | - | 13 | 4 | 3 | - | 20 |
| F1-186 | - | 18 | 1 | - | - | 19 |
| F5-127 | - | 14 | 4 | 1 | - | 19 |
| F4-2 | - | 18 | - | - | - | 18 |
| F5-25 | - | 18 | - | - | - | 18 |
| F3-3 | - | 15 | 2 | - | - | 17 |
| F5-69 | 3 | 11 | 3 | - | - | 17 |
| F5-8 | - | - | 16 | - | - | 16 |
| F6-7 | - | - | - | 16 | - | 16 |
| F3-56 | - | 14 | - | - | - | 14 |
| F1-81 | - | 12 | 1 | 1 | - | 14 |
| F1-173 | - | 11 | - | 3 | - | 14 |
| F1-35 | - | 11 | 3 | - | - | 14 |
| F4-1 | - | - | 14 | - | - | 14 |
| F1-89 | - | 12 | - | - | - | 12 |
| F3-43 | - | 10 | 1 | 1 | - | 12 |
| F5-101 | - | 11 | - | - | - | 11 |
| F5-140 | - | 11 | - | - | - | 11 |
| F6-6 | - | 9 | - | 2 | - | 11 |
| F1-28 | - | 10 | - | - | - | 10 |
| F3-18 | - | 10 | - | - | - | 10 |
| F5-77 | - | 10 | - | - | - | 10 |
| F1-197 | - | 9 | - | - | - | 9 |
| F5-24 | - | 8 | - | - | 1 | 9 |
| F4-51 | 2 | 7 | - | - | - | 9 |
| F1-22 | - | 8 | - | - | - | 8 |
| F3-12 | - | 8 | - | - | - | 8 |
| F5-149 | - | 5 | - | 3 | - | 8 |
| F5-9 | - | 3 | 5 | - | - | 8 |
| F1-192 | - | 7 | - | - | - | 7 |
| F2-6 | - | 7 | - | - | - | 7 |
| F5-70 | - | 7 | - | - | - | 7 |
| F2-14 | - | 6 | 1 | - | - | 7 |
| F4-38 | - | 6 | - | - | 1 | 7 |
| F5-87 | - | 6 | 1 | - | - | 7 |
| F1-174 | - | 1 | - | 6 | - | 7 |
| F1-170 | - | 6 | - | - | - | 6 |
| F1-172 | - | 6 | - | - | - | 6 |
| F1-5 | - | - | 6 | - | - | 6 |
| F2-32 | - | 5 | 1 | - | - | 6 |
| F7-2 | 2 | 2 | - | 2 | - | 6 |
| F1-20 | - | 5 | - | - | - | 5 |
| F1-209 | - | 5 | - | - | - | 5 |
| F6-4 | - | 5 | - | - | - | 5 |
| F1-55 | - | - | 5 | - | - | 5 |
| F1-183 | - | 4 | 1 | - | - | 5 |
| F3-28 | - | 4 | 1 | - | - | 5 |
| F5-17 | - | 4 | - | 1 | - | 5 |
| F5-71 | - | 4 | - | - | 1 | 5 |
| F5-132 | - | 2 | - | 3 | - | 5 |
| F1-177 | - | 4 | - | - | - | 4 |
| F1-51 | - | 4 | - | - | - | 4 |
| F1-80 | - | 4 | - | - | - | 4 |
| F1-96 | - | 4 | - | - | - | 4 |
| F5-137 | - | 4 | - | - | - | 4 |
| F5-145 | - | 4 | - | - | - | 4 |
| F5-59 | - | 4 | - | - | - | 4 |
| F1-189 | 1 | 3 | - | - | - | 4 |
| F2-9 | - | - | 4 | - | - | 4 |
| F1-47 | - | 3 | 1 | - | - | 4 |
| F5-91 | - | 3 | - | - | 1 | 4 |
| F5-4 | - | 2 | 2 | - | - | 4 |
| F1-152 | - | 1 | 3 | - | - | 4 |
| F11-3 | 3 | 1 | - | - | - | 4 |
| F1-176 | - | 3 | - | - | - | 3 |
| F1-181 | - | 3 | - | - | - | 3 |
| F1-182 | - | 3 | - | - | - | 3 |
| F1-184 | - | - | 3 | - | - | 3 |
| F1-187 | - | 3 | - | - | - | 3 |
| F1-37 | - | 3 | - | - | - | 3 |
| F1-52 | - | 3 | - | - | - | 3 |
| F2-31 | - | 3 | - | - | - | 3 |
| F5-142 | - | 3 | - | - | - | 3 |
| F11-7 | 2 | - | - | 1 | - | 3 |
| F5-146 | - | 3 | - | - | - | 3 |
| F3-6 | - | - | 3 | - | - | 3 |
| F3-9 | - | - | 3 | - | - | 3 |
| F5-95 | - | 3 | - | - | - | 3 |
| F1-196 | - | 2 | - | - | 1 | 3 |
| F5-134 | - | 2 | 1 | - | - | 3 |
| F11-1 | 2 | 1 | - | - | - | 3 |
| F1-158 | - | 2 | - | - | - | 2 |
| F1-175 | - | 2 | - | - | - | 2 |
| F1-178 | - | 2 | - | - | - | 2 |
| F1-18 | - | - | 2 | - | - | 2 |
| F1-190 | - | - | 2 | - | - | 2 |
| F1-199 | - | 2 | - | - | - | 2 |
| F1-2 | - | - | 2 | - | - | 2 |
| F1-200 | - | 2 | - | - | - | 2 |
| F1-64 | - | - | 2 | - | - | 2 |
| F1-90 | - | 2 | - | - | - | 2 |
| F2-12 | - | 2 | - | - | - | 2 |
| F11-5 | - | - | - | 2 | - | 2 |
| F11-8 | - | - | - | 2 | - | 2 |
| F2-13 | - | 2 | - | - | - | 2 |
| F3-59 | - | 2 | - | - | - | 2 |
| F4-57 | - | 2 | - | - | - | 2 |
| F4-63 | - | 2 | - | - | - | 2 |
| F4-64 | - | 2 | - | - | - | 2 |
| F4-60 | - | - | 2 | - | - | 2 |
| F4-65 | - | 2 | - | - | - | 2 |
| F4-66 | - | 2 | - | - | - | 2 |
| F5-143 | - | 2 | - | - | - | 2 |
| F5-151 | - | 2 | - | - | - | 2 |
| F5-154 | - | 2 | - | - | - | 2 |
| F5-155 | - | 2 | - | - | - | 2 |
| F5-160 | - | 2 | - | - | - | 2 |
| F11-2 | - | 1 | - | 1 | - | 2 |
| F3-13 | - | 1 | 1 | - | - | 2 |
| F5-19 | - | - | 2 | - | - | 2 |
| F1-119 | - | - | - | 1 | - | 1 |
| F1-15 | - | - | 1 | - | - | 1 |
| F1-157 | - | 1 | - | - | - | 1 |
| F1-159 | - | 1 | - | - | - | 1 |
| F1-167 | - | 1 | - | - | - | 1 |
| F1-169 | 1 | - | - | - | - | 1 |
| F1-171 | - | 1 | - | - | - | 1 |
| F1-179 | - | 1 | - | - | - | 1 |
| F1-180 | - | 1 | - | - | - | 1 |
| F1-185 | - | 1 | - | - | - | 1 |
| F1-188 | - | 1 | - | - | - | 1 |
| F1-19 | - | 1 | - | - | - | 1 |
| F1-191 | - | 1 | - | - | - | 1 |
| F1-193 | - | - | - | - | 1 | 1 |
| F1-194 | - | 1 | - | - | - | 1 |
| F1-195 | - | 1 | - | - | - | 1 |
| F1-198 | - | 1 | - | - | - | 1 |
| F1-201 | - | 1 | - | - | - | 1 |
| F1-202 | - | 1 | - | - | - | 1 |
| F1-203 | - | 1 | - | - | - | 1 |
| F1-204 | - | 1 | - | - | - | 1 |
| F1-205 | - | 1 | - | - | - | 1 |
| F1-206 | - | 1 | - | - | - | 1 |
| F1-207 | - | 1 | - | - | - | 1 |
| F1-208 | - | 1 | - | - | - | 1 |
| F1-33 | - | 1 | - | - | - | 1 |
| F1-68 | - | 1 | - | - | - | 1 |
| F1-94 | - | - | 1 | - | - | 1 |
| F1-99 | - | 1 | - | - | - | 1 |
| F10-1 | - | 1 | - | - | - | 1 |
| F11-6 | - | 1 | - | - | - | 1 |
| F2-33 | - | 1 | - | - | - | 1 |
| F2-7 | - | 1 | - | - | - | 1 |
| F3-5 | - | - | 1 | - | - | 1 |
| F3-61 | - | - | - | 1 | - | 1 |
| F3-62 | - | 1 | - | - | - | 1 |
| F3-63 | - | 1 | - | - | - | 1 |
| F3-64 | - | - | 1 | - | - | 1 |
| F3-65 | - | 1 | - | - | - | 1 |
| F3-66 | - | - | 1 | - | - | 1 |
| F4-12 | - | 1 | - | - | - | 1 |
| F4-21 | - | - | 1 | - | - | 1 |
| F4-24 | - | 1 | - | - | - | 1 |
| F4-25 | - | 1 | - | - | - | 1 |
| F4-58 | - | 1 | - | - | - | 1 |
| F4-59 | - | - | 1 | - | - | 1 |
| F4-61 | - | 1 | - | - | - | 1 |
| F4-62 | - | 1 | - | - | - | 1 |
| F5-115 | - | 1 | - | - | - | 1 |
| F5-118 | - | - | 1 | - | - | 1 |
| F5-13 | - | - | 1 | - | - | 1 |
| F5-131 | - | 1 | - | - | - | 1 |
| F5-139 | - | 1 | - | - | - | 1 |
| F5-141 | - | 1 | - | - | - | 1 |
| F5-144 | - | 1 | - | - | - | 1 |
| F5-147 | - | 1 | - | - | - | 1 |
| F5-150 | - | 1 | - | - | - | 1 |
| F5-152 | - | 1 | - | - | - | 1 |
| F5-153 | - | - | 1 | - | - | 1 |
| F5-156 | - | 1 | - | - | - | 1 |
| F5-157 | - | 1 | - | - | - | 1 |
| F5-158 | - | 1 | - | - | - | 1 |
| F5-159 | - | 1 | - | - | - | 1 |
| F5-161 | - | 1 | - | - | - | 1 |
| F5-162 | - | 1 | - | - | - | 1 |
| F5-163 | - | 1 | - | - | - | 1 |
| F5-164 | - | 1 | - | - | - | 1 |
| F5-165 | - | 1 | - | - | - | 1 |
| F5-166 | - | 1 | - | - | - | 1 |
| F5-2 | - | - | 1 | - | - | 1 |
| F5-67 | - | 1 | - | - | - | 1 |
| F5-68 | - | - | 1 | - | - | 1 |
| F5-81 | - | 1 | - | - | - | 1 |
| F6-5 | - | - | 1 | - | - | 1 |
| F7-4 | - | - | - | 1 | - | 1 |
| Total | 113 | 2588 | 1683 | 290 | 24 | 4698 |

**Table S**6: Frequency of the most common meningococci strain types

| meningo_strain_type | frequency |
| --- | --- |
| *cnl*:P1.18-11,42-1:*fnl* | 438 |
| W:P1.5,2:F1-1 | 432 |
| W:P1.5-1,2-36:F5-1 | 105 |
| A:P1.20,9:F3-1 | 38 |
| *cnl*:P1.18-11,42-1:ND | 36 |
| W:P1.5,2:F6-3 | 25 |
| Y:P1.5-1,10-62:F1-3 | 24 |
| *cnl*:P1.18-11,ND:*fnl* | 24 |
| ND:P1.18-11,42-1:*fnl* | 23 |
| *cnl*:P1.ND,ND:ND | 23 |
| X:P1.5-1,10-1:F4-23 | 21 |
| Y:P1.5-1,10-8:F1-3 | 21 |

**Supplementary Figures**

**Supplemental Figure 1. Prevalence of *Neisseria* species carriage per survey, for each country**


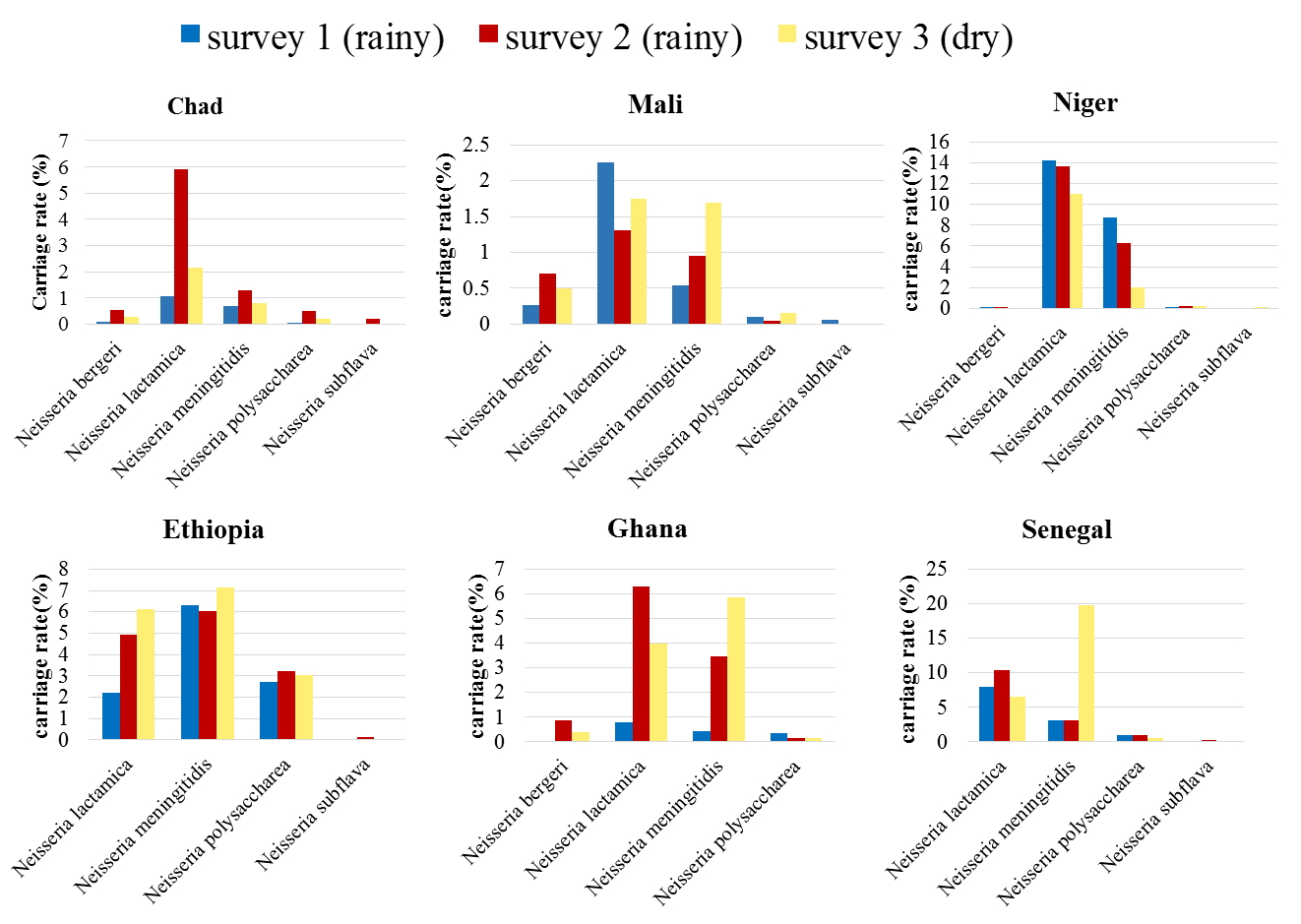


**Supplemental figure 2. Neisseria species carriage rate pre and post vaccine administration**


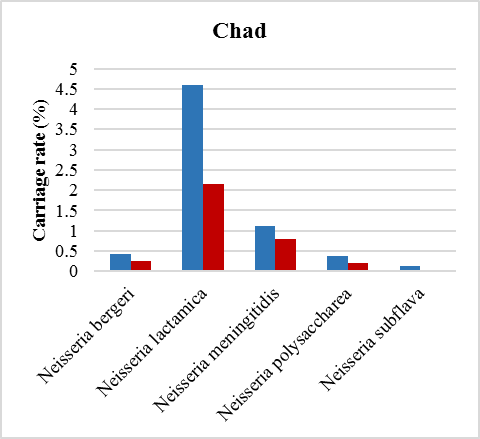

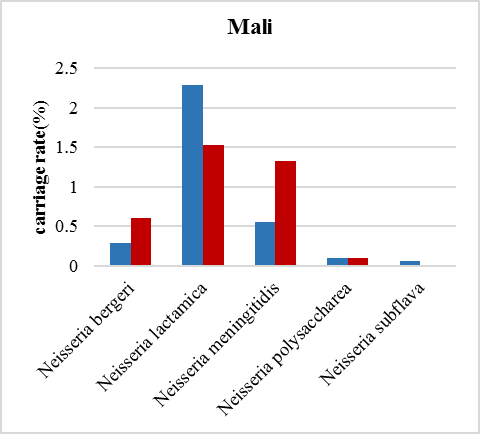

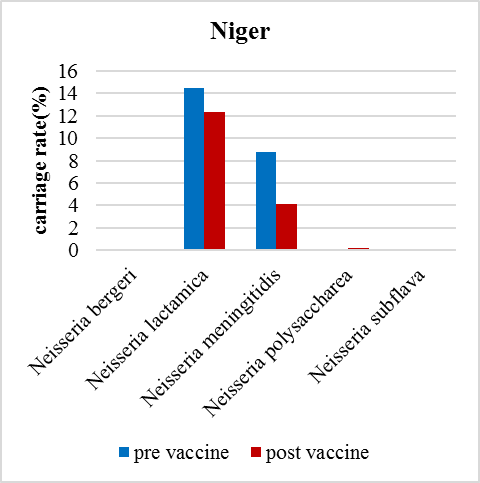

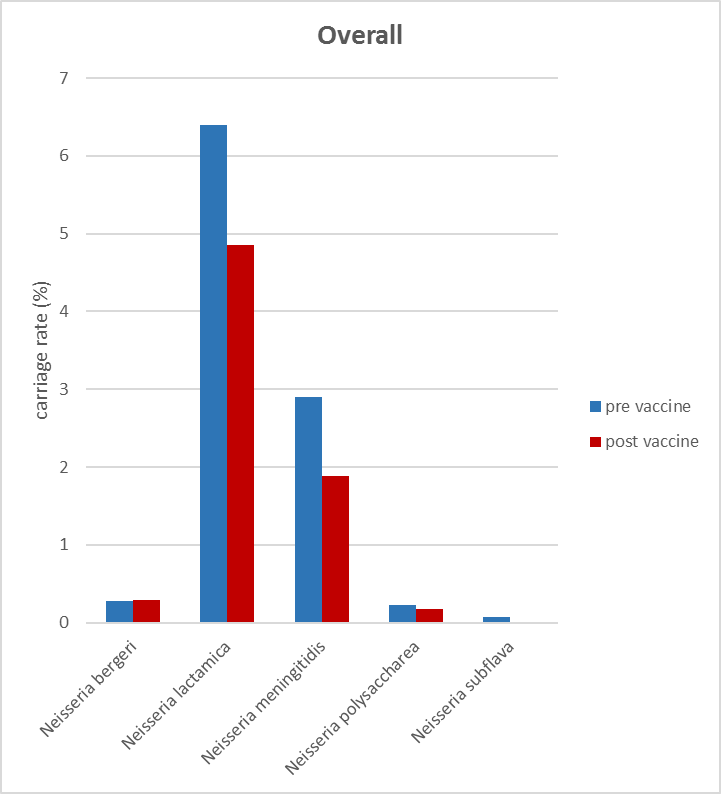


**D**

**C**

**A**

**B**

**Supplemental Figure 3: Evolutionary relationship of the *Neisseria* isolates based on *f_rplF***

Neighbor Joining tree representing the phylogeny based on the different *f_rplF* allele. The colors correspond to each species covered in this study: *Nm* (red),  *Nl* (yellow), *Np*  (purple), *Nb*  (blue) and *Ns* (orange). The circles correspond to the isolates used in the Bennett et al (2014) paper and the squares to the isolates described in this current work.

See attached pdf
