# Supplementary figures and images for: Pharyngeal carriage of Neisseria species in the African meningitis belt
Source: J Infect. 2016 Jun;72(6):667–77. doi: 10.1016/j.jinf.2016.03.010 (PMC4879866; doi:10.1016/j.jinf.2016.03.010)

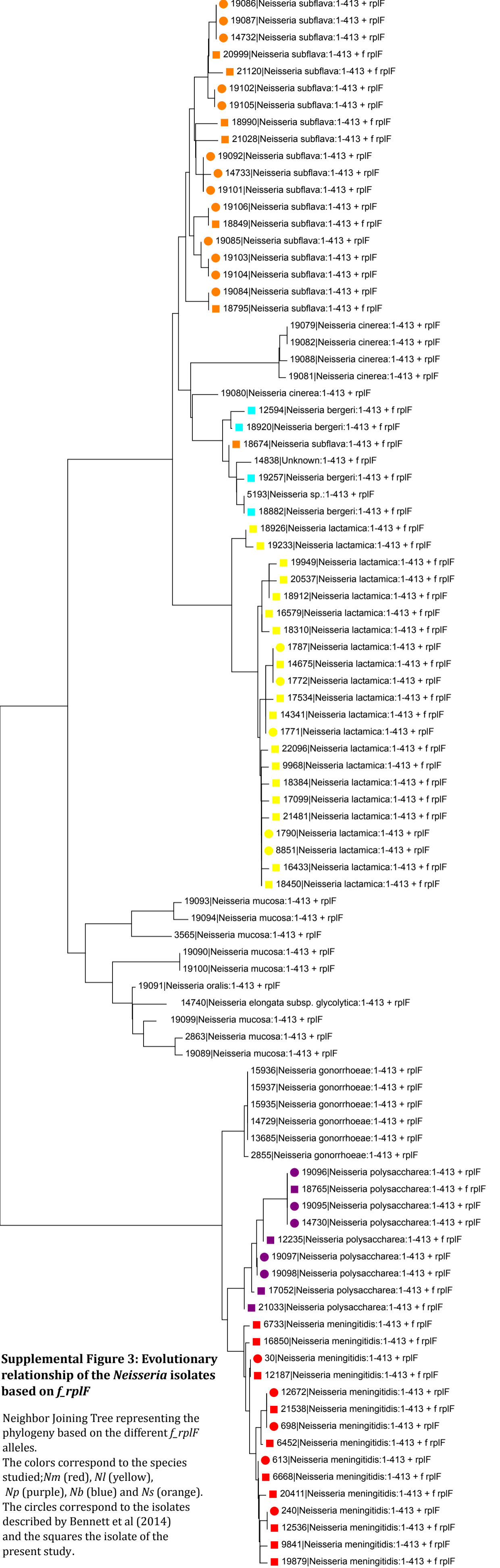

Supplement: Supplementary file 2 [file mmc2.pdf]
